# Elucidating the Solubility Enhancement of Active Pharmaceutical Ingredients through Hydrotropy: A Case of Local Anesthetics

**DOI:** 10.1021/acs.molpharmaceut.5c00628

**Published:** 2025-07-04

**Authors:** Sahar Nasrallah, Alexander Wendler, Sebastian A. Hallweger, Gregor Kieslich, Mirjana Minceva

**Affiliations:** † Biothermodynamics, TUM School of Life Sciences, 9184 Technical University of Munich, Maximus-von Imhof-Forum 2, Freising 85354, Germany; ‡ Department of Chemistry, TUM School of Natural Sciences, Technical University of Munich, Lichtenbergstraße 4, Garching 85748, Germany

**Keywords:** drugs solubility, solid−liquid equilibria, hydrotropy, eutectic mixtures, thermodynamic modeling

## Abstract

Hydrotropy has emerged as a promising approach to enhance the solubility and the availability of hydrophobic active pharmaceutical ingredients (APIs). To understand the hydrotropic effect on API solubility, it is crucial to investigate the molecular interactions and phase behavior in the API-hydrotrope-water system. The solid–liquid equilibrium (SLE) phase diagram of the ternary system aids in quantifying the hydrotropic effect and can guide the selection of an effective hydrotrope and its concentration. However, experimental determination of the complete SLE phase diagram at different temperatures is challenging and labor-intensive. This study introduces a thermodynamic-based method for selecting hydrotropes to enhance the solubility of APIs in water, considering API-hydrotrope, API-water, and hydrotrope-water interactions. The approach was demonstrated using three APIs, lidocaine, procaine, and benzocaine, and three hydrotropes, nicotinamide, caffeine, and urea. The SLE phase diagram of the ternary API-hydrotrope-water systems was predicted using the melting properties of the system components and their activity coefficients in the liquid solution, calculated with the nonrandom two-liquid (NRTL) model. The NRTL model binary interaction parameters were obtained from experimental SLE data for API-hydrotrope, API-water, and hydrotrope-water binary systems. The predicted SLE diagrams of the ternary API-hydrotrope-water systems revealed that the studied systems are eutectic systems with maximum API solubility at the eutectic point. Moreover, the thermodynamic analysis has shown that an efficient hydrotrope strongly interacts with API and water, with nicotinamide yielding the highest API solubility enhancement for the studied systems. This study highlights the potential of thermodynamic modeling in guiding the selection of hydrotropes and their concentrations to achieve the targeted API solubility in water.

## Graphical abstract

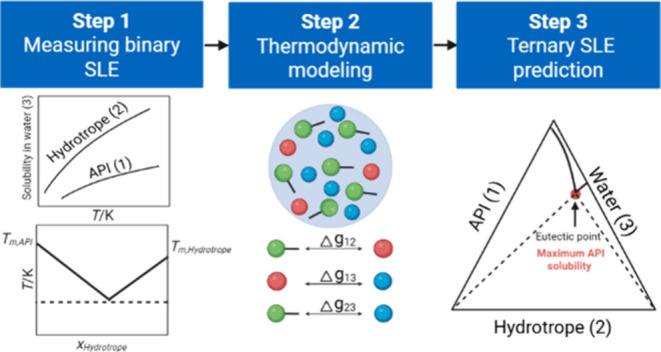

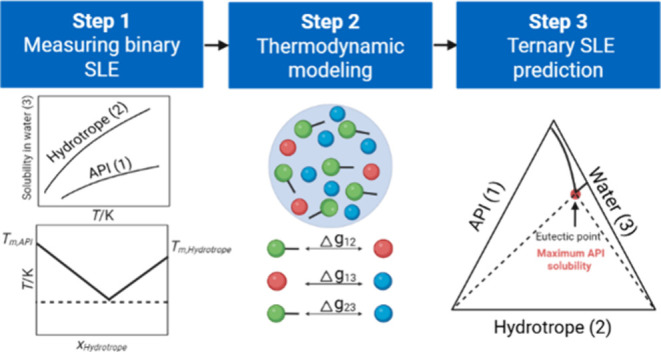

## 1 Introduction

The development and design of new pharmaceutical drugs require understanding the solubility of active pharmaceutical ingredients (APIs) when combined with excipients. Excipients are inert substances added to APIs to improve their long-term stability, solubility, bioavailability, and therapeutic efficacy.^1^ The hydrophobic nature of many APIs limits their solubility in water, often resulting in low bioavailability and reduced therapeutic efficacy. Hydrotropes are excipients used to improve API solubility in water.^2^ Hydrotropes are amphiphilic compounds that increase the solubility of hydrophobic substances in water through mechanisms other than micellar solubilization.^2–6^ For example, hydrotropes such as urea, sodium salicylate, sodium acetate, and sodium benzoate have been shown to enhance the solubility of different APIs, including aceclofenac, diclofenac sodium, furosemide, hydrochlorothiazide, and rifabutin, by factors ranging from 6 to over 2600-fold.^6–13^

Hydrotropes improve API solubility by creating a favorable microenvironment around the API molecules.^14^ The hydrotropic mechanisms include self-aggregation, disruption of water structure, complex formation with the API, or a combination of these mechanisms.^4,15^ However, these mechanisms lack a solid thermodynamic foundation and are not fully understood.^16^ Shimizu et al. demonstrated that the stoichiometric binding model fails to capture preferential drug–hydrotrope interactions and, therefore, cannot fully explain the mechanisms of hydrotropy.^16,17^ They employed the fluctuation solution theory (FST),^17^ originally developed by Kirkwood and Buff (KB),^18^ to analyze drug–hydrotrope interactions and hydrotrope self-association directly from thermodynamic data, including drug solubility in hydrotropic solutions, water activity coefficients, and density data.^16,19,20^ The hydrotrope-solute interactions in hydrotrope-solute-water solutions have been studied using other methods, such as NMR spectroscopy.^21,22^

Given the limited understanding of hydrotropic mechanisms and the wide variety of potential hydrotropes with diverse molecular structures, their selection is typically made through labor-intensive experimental trial-and-error screening procedures. API solubility in water-hydrotrope solutions is usually measured using the isothermal shake flask method.^23^ In this method, an excess amount of API is added to the aqueous solution to ensure saturation, resulting in the formation of a solid precipitate. The API solubility is then determined at a constant temperature in the presence of different amounts of hydrotrope and plotted against the hydrotrope concentration in the solution. This method assumes that the API reaches its maximum solubility when its concentration in solution no longer increases, even with further addition of hydrotrope. Additionally, it is assumed that the precipitate consists solely of the API, which may not always be valid, particularly when API complex formation or coprecipitation with hydrotrope occurs. The precipitated solid phase can be confirmed by Powder X-ray Diffraction (PXRD) analysis. The shake flask method has several disadvantages, including the need for extensive analytical techniques to measure the concentrations of both API and hydrotropes. Furthermore, the method typically requires 48 to 72 h for equilibration, during which time the API may undergo degradation or polymorphism changes, potentially compromising the accuracy of the obtained results.

To select the appropriate hydrotrope and its concentration for achieving the targeted API solubility, determining the solid–liquid equilibrium (SLE) of the API-hydrotrope-water system is essential. The SLE provides information on both the solubility of the API and the crystallized solid phase at a specific temperature. However, experimental solubility measurements of APIs in ternary API-hydrotrope-water mixtures and the determination of the complete SLE phase diagram across all compositions and/or at different temperatures are time-consuming and resource-intensive. Thermodynamic models can be used to predict the SLE phase diagram at any temperature, thereby reducing the need for extensive experimental work. Thermodynamic modeling of solubility requires knowledge of the melting properties of the API, hydrotrope, and water, as well as their activity coefficients in the liquid phase.^24^ The activity coefficients of components in the liquid phase quantify the intermolecular interactions between the API, hydrotrope, and water. Thermodynamic models, such as activity coefficient models, can be used to describe the nonideality of the liquid solution and calculate the activity coefficients of the components in the liquid phase. These models are typically classified into three categories: correlative, predictive, or semipredictive. Correlative models, such as Wilson,^25^ Van Laar,^26^ Two-Suffix Margules,^26^ NRTL,^27^ and UNIQUAC,^28^ utilize experimental equilibrium data to fit adjustable parameters and calculate solubility without explicitly relying on the molecular structure of the components. Predictive models, including UNIFAC,^29^ and COSMO-RS,^30^ estimate solubility based on molecular structure and theoretical principles, thereby reducing reliance on experimental data. Semipredictive models, such as PC-SAFT,^31^ and NRTL-SAC,^32^ integrate aspects of both approaches by combining structural information with limited experimental input.

The influence of excipient melting properties and activity coefficients on the API-excipient binary SLE has been extensively studied in the literature.^33–36^ However, evaluating the ability of a hydrotrope to enhance API solubility in water requires considering the solvent effect, including interactions among API-hydrotrope, API-water, and hydrotrope-water. In our prior theoretical studies,^37,38^ we investigated the influence of excipient (hydrotrope) melting properties, the pairwise interactions between API-excipient, API-solvent, and excipient-solvent, and the temperature on API solubility enhancement. We analyzed their impact on the shape of solubility isotherms for hypothetical ternary API-excipient-water systems to identify the composition with maximum API solubility. In Nasrallah et al.,^37^ we considered the case of excipient melting temperature below the system (solution) temperature, meaning that the excipient is liquid at the system temperature. In this case, the excipient does not exhibit a solubility line, and the ternary system does not form a eutectic system. In our second study, Nasrallah et al.,^38^ the excipient was assumed to be in the solid phase at the solution temperature (i.e., the excipient melting temperature higher than the ternary system temperature). In this case, both the API and the excipient have solubility lines, and the ternary mixtures form a eutectic system with a eutectic point at the intercept of the API and excipient solubility lines. Our findings indicated that the maximum API solubility is achieved at the eutectic point of the API-excipient-water system. The formation of the eutectic point in the ternary API-excipient-water systems is rarely analyzed in hydrotropy studies, as the full ternary SLE diagram is not usually measured.

In this work, we aim to explore hydrotropy from a thermodynamic perspective by analyzing the binary interactions between the API, hydrotrope, and water. Combining experimental binary SLE data with thermodynamic modeling, we quantify the binary interaction parameters and predict the ternary SLE phase diagram. We propose a thermodynamic model-based approach to predict the SLE phase diagram of a ternary API-hydrotrope-water system for hydrotropes that, in their pure form, are solid at the regarded system temperature. The API-hydrotrope-water SLE diagrams should serve the selection of a hydrotrope and its concentration for achieving a targeted API solubility. Lidocaine, procaine, and benzocaine—a widely used local anesthetics for pain relief in minor injuries and dental care—were selected due to their therapeutic relevance and poor water solubility together with the commonly used hydrotropes: nicotinamide, caffeine, and urea. First, the solubility (SLE) of the model APIs and hydrotropes in water was measured at different temperatures using the temperature-variant method. Then, the SLE of the API-hydrotrope systems was measured using differential scanning calorimetry (DSC). The binary systems SLE data were then used to obtain the binary interaction parameters of the NRTL model. After that, the SLE phase diagram of the ternary API-hydrotrope-water system was calculated at the basal human body temperature and used to identify the best hydrotrope and the maximum solubility of the API in the ternary system. Lastly, the practical application of the predicted SLE phase diagrams for product formulation is discussed.

## 2 Materials and Method

### 2.1 Chemicals

Nicotinamide (≥99%), caffeine (≥99%), urea (≥99%), lidocaine (≥99%), and benzocaine (≥99%) were purchased from Sigma-Aldrich (Sigma-Aldrich, St. Louis, USA). Procaine (≥99%) was bought from BSDpharm (BSDpharm, USA). HPLC-grade water was purchased from VWR Chemicals (VWR International, Radnor, USA). All chemicals were used as received without purification. The molecular structures of the studied APIs and hydrotropes are provided in Figure 1.

**Figure 1:**
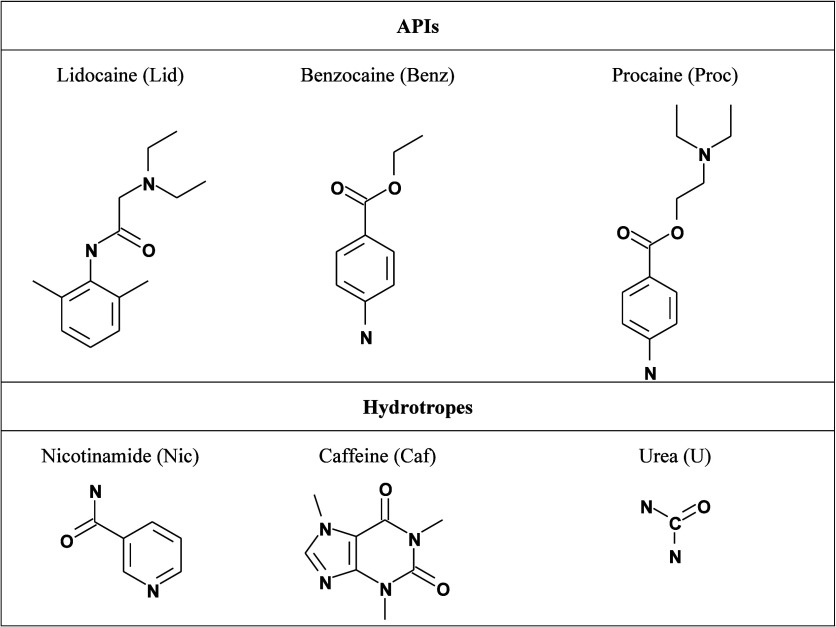
Molecular structures of the studied local anesthetics (APIs) and hydrotropes.

### 2.2 Powder X-ray Diffraction

PXRD was used to characterize the crystalline solid phases of the pure component (APIs and hydrotropes) and the binary API-hydrotrope mixture at a 1:1 molar ratio. The samples were packed into borosilicate glass capillaries (Hilgenberg, Germany) with a 1.5 mm diameter after 20 min grinding at room temperature.

PXRD measurements were performed at ambient temperature using Debye–Scherrer geometry in an STOE Stadi P diffractometer with a DECTRIS Multi-MYTHEN detector, a curved Ge(111) monochromator, and a Mo Kα̲ radiation source (λ = 0.70926 Å). Data were collected over a 2θ range of 2° to 39.55° with a step size of 0.015° (2θ). Two ranges were measured and subsequently summed to improve the signal-to-noise ratio The phase purity of the purchased pure compounds was verified by comparing measured PXRD with calculated patterns from single-crystal X-ray diffraction or neutron diffraction data.^39–45^ The calculated PXRD patterns were generated using the program VESTA.^46^ Minor deviations from the calculated patterns can be attributed to thermal expansion effects caused by differences in the measurement temperatures of the literature data and the capillary measurements. The PXRD patterns of the three APIs and three hydrotropes at ambient temperature, compared to calculated patterns, are shown in Figures S1–S6 in the Supporting Information file. The PXRD pattern of the binary mixtures of the APIs and hydrotropes (1:1 molar ratio), compared to pure APIs and hydrotropes at ambient temperature, are shown in Figures S7–S15 in the Supporting Information file.

We would like to point out that benzocaine exhibits three different polymorphic structures at ambient conditions.^43,44^ We observed that the polymorphic composition changes upon grinding, which can be described by a nearly quantitative phase transition from polymorph III^44^ to polymorph II.^43^ Comparison with the calculated PXRD patterns of the polymorphs, as well as a multiphase Pawley fit analysis, suggests a phase mixture in the purchased benzocaine of polymorphs II and III. The PXRD patterns of benzocaine (as purchased after 20 min of grinding and calculated patterns of the three polymorphs—polymorph I,^44^ polymorph II,^43^ and polymorph III^44^) are shown in Figure S16 in the Supporting Information file. The Pawley profile of the purchased benzocaine indicates a phase mixture consisting of polymorphs II and III before grinding, which is shown in Figure S17 in the Supporting Information file. After 20 min of grinding, the PXRD shows that the majority phase of the sample is polymorph II, as shown in Figure S18 in the Supporting Information file.

### 2.3 Differential Scanning Calorimetry

DSC analysis was used to obtain the melting properties of the pure components (APIs and hydrotropes) and the SLE data for the nine binary systems, each consisting of one API (benzocaine, lidocaine, or procaine) and one hydrotrope (nicotinamide, caffeine, or urea). Samples were weighed using a Sartorius analytical balance (Germany) with an uncertainty of ±0.01 mg for both pure components and binary mixtures.

Binary mixtures were prepared in various ratios (0.1–0.9 mole fraction), sealed in glass vials, and heated under continuous mixing until a clear liquid formed. The samples were stored at 253 K to ensure complete crystallization before analysis, and the crystallized solids were ground to a fine, homogeneous powder using a mortar and pestle. For the binary systems containing caffeine, the components were ground directly without prior melting due to caffeine’s high melting temperature.

The DSC instrument was calibrated using six reference standards (adamantane, indium, tin, zinc, bismuth, and cesium chloride) at a heating rate of 5 K min^–1^. Measurements were performed in an inert environment using nitrogen with a flow rate of 150 mL min^–1^. For each binary mixture consisting of an API and a hydrotrope, the DSC sample (4–6 mg of the ground powder) was hermetically sealed and heated at a rate of 5 K min^–1^ to a maximum temperature of 521 K for caffeine systems, 411 K for nicotinamide systems, and 416 K for urea systems, selected based on their melting points to ensure complete melting without thermal degradation. The solidus transition and liquidus temperatures were determined as the onset and peak maximum temperatures, respectively. For pure components, melting temperatures (*T*_m_) and melting enthalpies (Δ*h*_m_) were obtained from the onset temperature and peak area, respectively. The DSC curves of pure APIs, pure hydrotropes, and the API-hydrotrope binary mixtures at the eutectic points are shown in Figures S19–S21 in the Supporting Information file, respectively.

### 2.4 Solubility Measurements

The solubility of pure APIs, hydrotropes, and API-hydrotrope mixtures in water was measured using a Crystal16 multireactor crystallizer (Technobis, Netherlands). This instrument determines the melting temperature of a solution by measuring light transmissivity through the sample, identifying the “clear point”—the temperature at which the solution becomes fully transparent at a known composition. Accurately weighed amounts of the solid phase were added to 1 mL of water in glass vials and then subjected to a thermal program under continuous stirring. The temperature was increased from 273.15 to 10 K below the boiling point of water at a rate of 0.5 K min^–1^. For pure APIs, due to their relatively low melting temperatures (see Table 1), the temperature was raised only up to a value just below their melting points. The clear point temperature was recorded as the liquidus temperature of the mixture at the given composition. All measurements were performed in triplicate.

### 2.5 Thermodynamic Modeling

The solubility curves in the phase diagrams were calculated using

$$\ln\;x_{i}^{L}\gamma_{i}^{L}=-\frac{\Delta h_{m,i}}{RT}\left(1-\frac{T}{T_{m,i}}\right)-\frac{\Delta h_{\mathrm{tr},i}}{RT}\left(1-\frac{T}{T_{\mathrm{tr},i}}\right)-\frac{\Delta c_{p,i}}{R}\left(1-\frac{T_{m,i}}{T}+\ln\;\frac{T_{m,i}}{T}\right)$$ 1

where *x*_*i*_^L^ and γ_*i*_^L^ are the mole fraction and activity coefficient of component *i* in the liquid phase, respectively; *T* is the temperature; Δ*h*_m,*i*_ and *T*_m,*i*_ are the melting enthalpy and temperature of pure component *i*, respectively; Δ*h*_tr,*i*_ and *T*_tr,*i*_ are the solid–solid transition enthalpy and temperature of pure component *i*, respectively; Δ*c*_p_ is the difference between the constant pressure heat capacity of pure component *i* in the solid and liquid states at *T*_m,*i*_; *R* is the universal gas constant. In many cases, the Δ*c*_p_ term (second term on the right-hand side) has a minor influence on the solubility curve compared to the Δ*h*_m,*i*_ term (first term on the right-hand side).^26^ Thus, for the sake of simplicity, Δ*c*_p_ was not considered a parameter in this study, and the following expression was used to calculate the solubility line:

$$\ln\;x_{i}^{L}\gamma_{i}^{L}=-\frac{\Delta h_{m,i}}{RT}\left(1-\frac{T}{T_{m,i}}\right)-\frac{\Delta h_{\mathrm{tr},i}}{RT}\left(1-\frac{T}{T_{\mathrm{tr},i}}\right)$$ 2

The melting properties of a pure component are influenced by molecular symmetry, conformational diversity, and intermolecular forces.^47^ At the melting point, the melting properties are interrelated by the following equation:

$$\Delta h_{m}=T_{m}\Delta s_{m}$$ 3

where Δ*s*_m_ is the melting entropy.

The activity coefficients of the components in the liquid phase were calculated using the NRTL model as follows:^27^

$$\ln(\gamma_{i}^{L})=\frac{\sum_{j=1}^{C}\tau_{ji}G_{ji}x_{j}}{\sum_{j=1}^{C}G_{ji}x_{j}}+\sum_{j=1}^{C}\frac{x_{j}G_{ij}}{\sum_{k=1}^{C}x_{k}G_{kj}}\left(\tau_{ij}-\frac{\sum_{k=1}^{C}x_{k}\tau_{kj}G_{kj}}{\sum_{k=1}^{C}x_{k}G_{kj}}\right)$$ 4

$$G_{ij}=\exp\left(-\alpha_{ji}\tau_{ij}\right),G_{ji}=\exp\left(-\alpha_{ji}\tau_{ji}\right)$$ 5

$$\tau_{ij}=\frac{\Delta g_{ij}}{RT},\tau_{ji}=\frac{\Delta g_{ji}}{RT}$$ 6

where Δ*g*_*ij*_ and Δ*g*_*ji*_ are the binary interaction parameters, and α_*ij*_ denotes the nonrandomness factor, assumed to equal 0.3. The binary interaction parameters were obtained by minimizing the following objective function:

$$\mathrm{OF}(T)=\sum_{i}^{n}{\left(\frac{(T_{i}^{\exp}-T_{i}^{\mathrm{cal}}{)}^{2}}{n}\right)}^{1/2}$$ 7

where *T*_*i*_^exp^ and *T*_*i*_^cal^ are the experimental and calculated liquidus temperatures, respectively, and *n* is the number of data points. The discrepancy between the calculated liquidus temperatures (*T*_*i*_^cal^) and the experimentally measured temperatures (*T*_*i*_^exp^) was assessed by calculating the root-mean-square deviation (RMSD) as follows:

$$\mathrm{RMSD}=\sqrt{\frac{\sum_{i}^{n}(T_{i}^{\exp}-T_{i}^{\mathrm{cal}}{)}^{2}}{n}}$$ 8

In systems exhibiting both solid–liquid equilibrium (SLE) and liquid–liquid equilibrium (LLE), such as the binary systems of lidocaine, benzocaine, and procaine with urea, calculating activity coefficients using the NRTL model is challenging, as it requires extensive experimental data to characterize LLE. Therefore, the predictive COSMO-RS model was used to calculate the activity coefficients of the APIs and urea in the liquid phase of their binary mixtures.

Molecular conformations of the APIs and urea were generated using COSMOconf17 (Dassault Systèmes, France, version 4.2), while molecular geometry optimization and screening charge density calculations were performed using Turbomole (TURBOMOLE GmbH, Germany, version 6.6). COSMO-RS calculations were carried out using COSMOtherm19 (Dassault Systèmes, France, version 19) with the BZ_TZVPD_FINE_19.ctd parameter sets. The binary interaction parameters for each API-urea system (lidocaine-urea, benzocaine-urea, and procaine-urea) were fitted to the activity coefficients obtained from COSMO-RS using the NRTL model.

### 2.6 Solubility Enhancement Factor

In this study, solubility (*S*) was defined as the moles of the API dissolved in moles of water at a specific temperature and calculated using the following equation:

$$S=\frac{x_{1}}{x_{3}}$$ 9

where *x*_1_ and *x*_3_ are the mole fraction of API and water, respectively. The API solubility enhancement due to the addition of hydrotrope was evaluated using the solubility enhancement factor (Φ), which was defined as the ratio between the API solubility in the presence of the hydrotrope and its solubility without the hydrotrope:^48^

$$\Phi =\frac{S_{x_{2}\neq 0}}{S_{x_{2}=0}}$$ 10

where *x*_2_ is the hydrotrope mole fraction. Φ can be calculated using eq 10 with the mole factions of components calculated with eq 2.

## 3 Results and Discussion

In this section, the results of the experimental measurements, thermodynamic modeling, and validation of the approach used to predict API solubility in API-hydrotrope-water systems are presented. In Section 3.1, the NRTL model binary interaction parameters are fitted to experimentally determined solubility of the APIs and the hydrotropes in water at different temperatures, along with the SLE data for binary API-hydrotrope. In Section 3.2, the calculated binary SLE phase equilibrium data are compared with the corresponding experimental data. In Section 3.3, the determined NRTL binary interaction parameters were further applied to predict the SLE phase diagram of the ternary API-hydrotrope-water systems at basal human body temperature, and the thermodynamic analysis of molecular interactions was used to explain how hydrotrope selection influences API solubility. In Section 3.4, the accuracy of the NRTL model predictions was validated against experimental data. Finally, in Section 3.5, the application of API-hydrotrope-water ternary SLE phase diagrams for selecting the API-hydrotrope molar ratio for preparing an aqueous solution with maximally achievable API solubility while preventing the crystallization of the API or hydrotrope due to temperature fluctuation—for example, during transportation or storage—is presented.

### 3.1 Determining NRTL Model Binary Interaction Parameters

The NRTL model was used to model the API-hydrotrope-water systems and select the best hydrotrope. The melting properties of the selected APIs and hydrotropes measured in this work are presented in Table 1. They are in accordance with the data published in the literature (Table S1 in the Supporting Information file), except for procaine, for which noticeable differences in melting properties have been reported by different research groups (see ref 9 in the Supporting Information file). Additionally, the melting entropies of the APIs and hydrotropes were calculated using eq 3 and are reported in Table 1.

**Table 1:** Melting Temperature (*T*_m_) and Enthalpy (Δ*h*_m_) of the Selected APIs and Hydrotropes, Measured in This Work, and Entropy (Δ*s*_m_) Calculated Using Eq 3

compound	*T*_m_/ K	Δ*h*_m_/kJ mol^‑1^	Δ*s*_m_/J mol^–1^ K^–1^
APIs			
lidocaine	340.86	18.75	55.01
benzocaine	362.32	25.03	69.08
procaine	333.38	29.48	88.43
hydrotropes			
nicotinamide	401.72	25.32	63.03
caffeine^a^	509.61	20.51	40.24
urea	405.85	14.80	36.47

a *T*_tr_ = 417.20 K and the Δ*h*_tr_ = 3.30 kJ mol^–1^.

The NRTL model binary interaction parameters were obtained by fitting the experimental phase equilibrium data of the API-hydrotrope, API-water, and hydrotrope-water binary systems. The SLE data for the binary API-hydrotrope systems, APIs solubility in water, and hydrotropes solubility in water data are provided in the Supporting Information file in Tables S2–S4, respectively. For mixtures containing urea, both LLE and SLE coexist, as explained in Section 2.5. Due to the absence of experimental LLE data, the activity coefficients of the components in the liquid phase were calculated using the COSMO-RS model within the temperature range corresponding to each experimental SLE data point. The binary interaction parameters of the NRTL model were then fitted to the activity coefficients obtained from COSMO-RS calculations.

The obtained NRTL model binary interaction parameters and calculated infinite dilution activity coefficients (lnγ^∞^) of components in the binary systems at 298.1 K are shown in Table 2. In this study, deviations from ideality are interpreted based on the values of infinite dilution activity coefficients (lnγ_*i*_^∞^). In binary systems consisting of components 1 and 2, lnγ_1_^∞^ represents the affinity of component 1 toward component 2, and vice versa lnγ_2_^∞^ represents the affinity of component 2 for component 1.^49,50^

**Table 2:** NRTL Model Binary Interaction Parameters and Infinite Dilution Activity Coefficient (lnγ^∞^) of Components Calculated at 298.1 K^a^

system	(1–2)	*g*_12_ (kJ mol^–1^)	*g*_21_ (kJ mol^–1^)	lnγ_1_^*∞*^ (−)	lnγ_2_^*∞*^ (−)	RMSD (K)
API + water	Benz-water	285.10	19.99	8.06	119.04	1.71
	Lid-water	298.19	23.06	9.30	120.86	2.35
	Pro-water	43.39	21.05	8.58	18.17	1.49
hydrotrope + water	Caf-water	–6.77	22.45	2.86	–2.13	3.31
	Nic-water	7.79	–4.29	–0.51	0.23	1.94
	U-water	–4.07	6.23	–0.17	–0.46	1.32
API + hydrotrope	Benz-Nic	3.53	–0.63	0.67	1.15	2.31
	Benz-Caf	–6.36	15.75	0.81	–1.62	2.72
	Lid-Nic	1.49	1.01	0.91	0.96	1.79
	Lid-Caf	–4.10	9.32	1.04	–0.44	4.43
	Pro-Nic	1.02	1.59	1.01	0.94	0.92
	Pro-Caf	–4.50	11.59	1.55	–0.66	3.52
	Benz-U	2.79	11.63	5.49	2.27	0.30
	Lid-U	14.35	19.99	9.08	6.51	0.30
	Pro-U	4.52	17.94	8.29	2.65	0.17

a The nonrandomness factor α_12_ = α_21_ and set to 0.3.

In API-water systems, a strong positive deviation from ideality was observed, indicated by the highly positive lnγ_1_^∞^ and lnγ_2_^∞^ values. This reflects unfavored API-water interactions. In hydrotrope-water systems, the lnγ_1_^∞^ values vary depending on the hydrotrope. Among the studied hydrotropes, urea, nicotinamide, and caffeine all exhibit self-association.^51–53^ For the caffeine-water system, the positive lnγ_1_^∞^ indicates a low affinity of caffeine for water, suggesting strong self-association of caffeine molecules.^54^ The nicotinamide–water system shows a negative lnγ_1_^∞^, indicating favorable nicotinamide–water interactions. The urea-water system exhibits near-ideal behavior, with lnγ_1_^∞^ close to zero.^55^ In API-hydrotrope systems, lnγ_1_^∞^ is positive. However, for APIs with nicotinamide and caffeine, the positive deviation from ideality is less significant than in the urea systems, indicating that APIs interact more favorably with nicotinamide and caffeine compared to urea. In API-caffeine systems, the lnγ_2_^∞^ values suggest that caffeine preferentially interacts with the APIs rather than self-associating.

### 3.2 SLE Phase Diagram of Binary Systems

In this section, the calculated solubility of the APIs and hydrotropes in water and the SLE phase diagrams of binary API-hydrotrope systems are presented and analyzed. Additionally, the solubility of the APIs and hydrotropes in water and the SLE phase diagrams of binary API-hydrotrope systems were also calculated, assuming ideal solution behavior (γ_*i*_^L^ = 1). The extent of deviation from ideality provides direct insight into the strength of interactions within the binary systems, as discussed in the previous section, using the infinite dilution activity coefficients lnγ_1_^∞^ and lnγ_2_^∞^. The variations in the solubility of the APIs and hydrotropes in water, as well as in the SLE of the API-hydrotrope systems, are due to the combined effect of their melting properties and activity coefficients in the liquid solution. This can be attributed to the molecular structures of the APIs and hydrotropes, as shown in Figure 1.

#### 3.2.1 API-Water Binary SLE

In Figure 2a the binary SLE phase diagrams for the API-water systems and in Figure 2b the phase diagrams for the hydrotrope-water systems, both calculated using the NRTL model are presented. These results demonstrate that the NRTL model successfully captures the liquid phase nonideality and provides a good description of the solubility of the APIs and hydrotropes in water.

**Figure 2:**
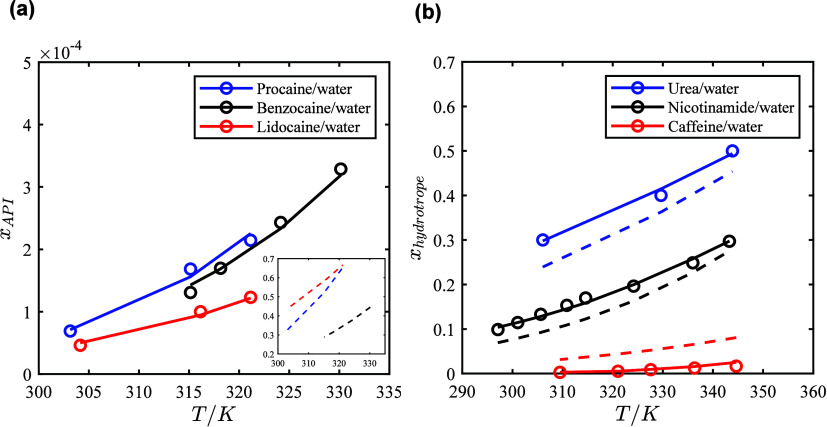
Solubility of (a) APIs in water at different temperatures and (b) hydrotropes in water at different temperatures. Circles—experimental data; lines—solubility calculated with the NRTL model; dashed lines—ideal solubility lines.

The solubility of the APIs in water calculated assuming ideal solubility behavior (γ_*i*_^L^ = 1) are significantly higher than the experimentally obtained solubilities shown in Figure 2a (see Figure S23a in the Supporting Information file) due to the highly positive deviation from ideality, as indicated by the lnγ_1_^∞^ and lnγ_2_^∞^ values (Table 2). The observed order of the ideal solubility lines of the APIs in water is primarily governed by APIs melting properties (Table 1). Lidocaine exhibits the highest ideal solubility, attributed to its lower melting temperature and enthalpy compared to the other APIs. Procaine, despite having a higher melting entropy and enthalpy compared to benzocaine, shows higher solubility due to its relatively lower melting temperature. The experimental solubility lines of the APIs in water follow a different order compared to the ideal solubility lines, which is due to their activity coefficients in the liquid solution. As shown in Figure 2a, the solubility of the APIs in water is extremely low, with procaine showing the highest solubility, followed by benzocaine with a similar solubility, while lidocaine exhibits the lowest solubility. Procaine’s amino group and alkylamino group (see Figure 1) allow for strong hydrogen bonding with water, while its ester group and aromatic ring contribute to its hydrophobic nature. Benzocaine’s amino group is capable of hydrogen bonding, but its more hydrophobic aromatic ring and ester group reduce its interaction strength with water compared to procaine. Lidocaine, with its aromatic ring and an amide group, has even weaker interactions with water relative to both procaine and benzocaine due to its lower polarity.

#### 3.2.2 Hydrotrope-Water Binary SLE

As seen in Figure 2b, the solubility of the hydrotropes in water, assuming ideal solution behavior (γ_*i*_^L^ = 1), follows the same order as their experimental solubility. This indicates that the variations in solubility are primarily due to the hydrotropes’ melting properties while the magnitude of solubility is driven by their interactions with water and the associated activity coefficients in the liquid solution. As shown in Figure 2b, urea and nicotinamide show a negative deviation from ideality, while caffeine shows a positive deviation as anticipated from the lnγ_1_^∞^ values (Table 2). Urea exhibits the highest solubility, followed by nicotinamide, with moderate solubility, while caffeine shows the lowest solubility. Urea, with its small, highly polar structure and multiple hydrogen bonding sites (see Figure 1), interacts strongly with water, resulting in its high solubility in water. Nicotinamide, with its aromatic ring and an amide functional group, has moderate hydrogen bonding potential, leading to intermediate solubility. In contrast, caffeine, with its rigid structure and multiple methyl groups, has fewer hydrogen bonding sites, reducing its affinity for water and leading to its lowest solubility in water among the three hydrotropes. The urea–water binary system, a well-studied reference in the literature, demonstrates that the calculated binary interaction parameters (see Table 2) successfully predict the urea–water SLE, which was calculated using IUPAC-fitted NRTL parameters based on extensive experimental data from the literature^56^ as shown in Figure S22 in the Supporting Information file. The solubility of the selected hydrotropes in water was compared with experimental data from the literature (nicotinamide,^57,58^ caffeine,^59^ and urea^56^), showing excellent agreement as illustrated in Figure S23b.

#### 3.2.3 API-Hydrotrope Binary SLE

The experimental and NRTL-modeled SLE phase diagrams of the binary API-hydrotrope systems for benzocaine, procaine, and lidocaine, each with the three selected hydrotropes, are presented in Figures 3, 4, and 5, respectively. As shown, the APIs and hydrotropes form eutectic mixtures, indicating preferred interactions between them. Based on the PXRD analysis, the comparison of the API–hydrotrope binary mixtures (1 mol API: 1 mol hydrotrope) with the pure APIs and hydrotropes showed that no cocrystallization or polymorphic transformation occurred during sample preparation, confirming the formation of API–hydrotrope physical mixtures without the appearance of new solid phases. DSC analysis further indicated that the mixtures of the APIs with nicotinamide and caffeine are completely miscible in the liquid phase and form eutectic mixtures, except for the API–urea mixtures, which exhibited only partial miscibility in the liquid phase.

**Figure 3:**
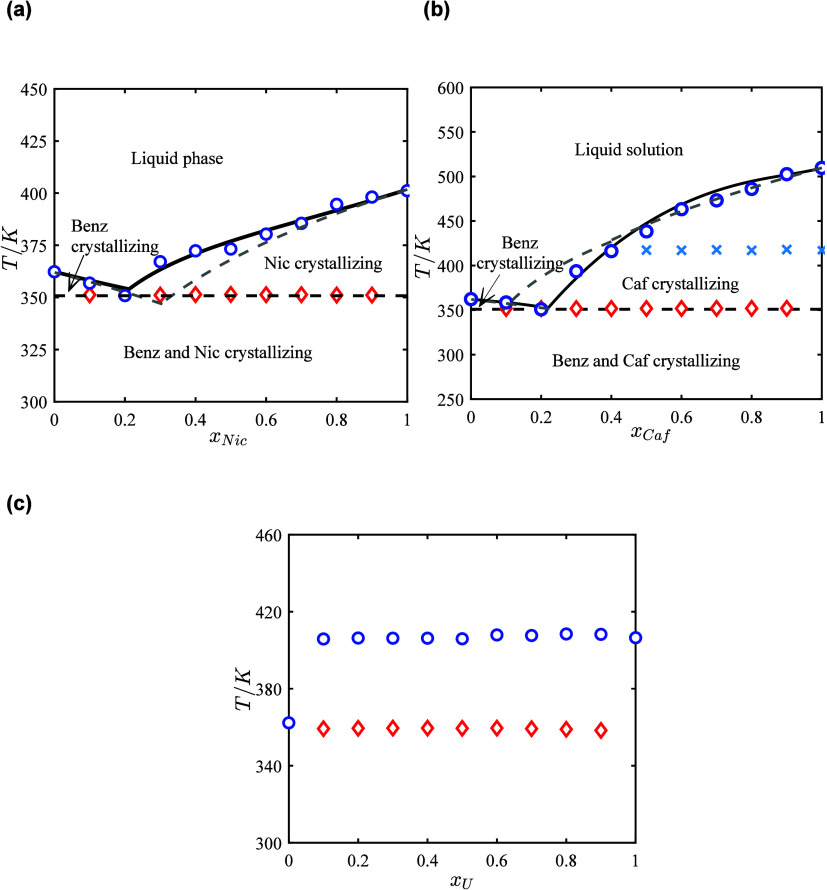
SLE phase diagrams of the benzocaine-hydrotrope systems: (a) benzocaine-nicotinamide, (b) benzocaine-caffeine, and (c) benzocaine-urea. Blue circles: liquidus temperatures; red diamonds: solidus (eutectic) temperatures. Solid black line: NRTL-modeled liquidus; dashed black line: NRTL-modeled solidus. Blue crosses: solid–solid transition temperature for caffeine. Dashed gray lines-ideal liquidus.

**Figure 4:**
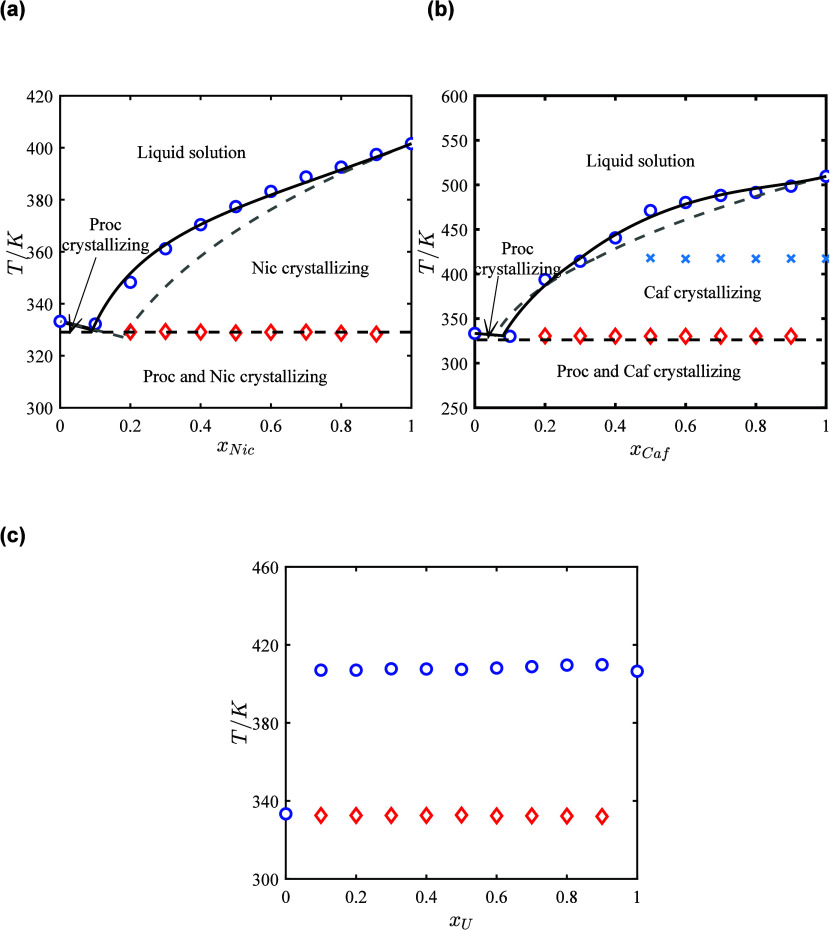
SLE phase diagrams of the procaine-hydrotrope systems: (a) procaine-nicotinamide, (b) procaine-caffeine, and (c) procaine-urea. Blue circles: liquidus temperatures; red diamonds: solidus (eutectic) temperatures. Solid black line: NRTL-modeled liquidus; dashed black line: NRTL-modeled solidus. Blue crosses: solid–solid transition temperature for caffeine. Dashed gray lines-ideal liquidus.

**Figure 5:**
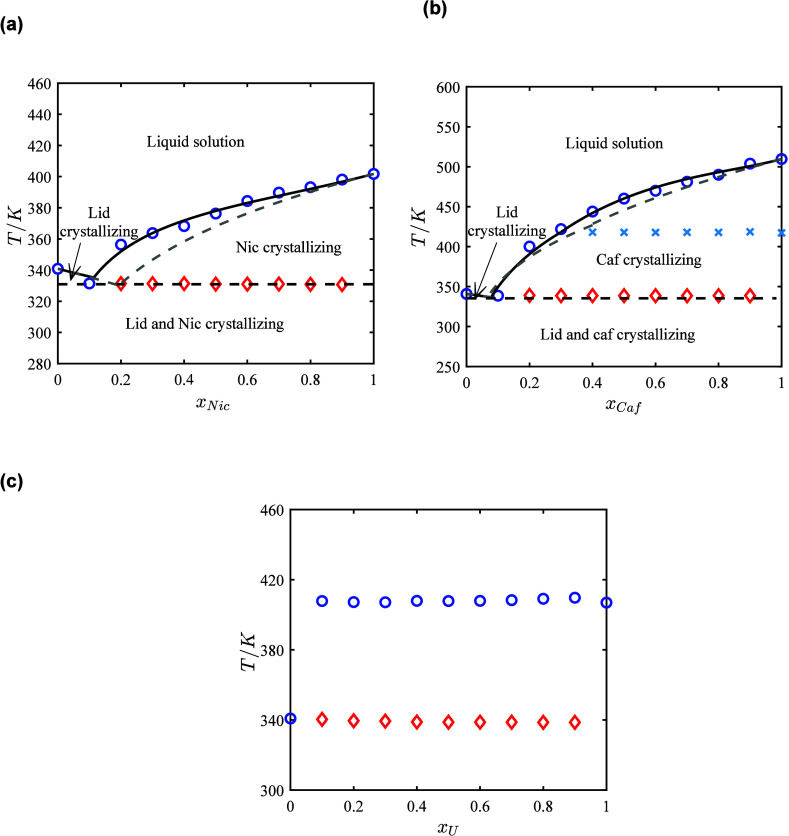
SLE phase diagrams of the lidocaine-hydrotrope systems: (a) lidocaine-nicotinamide, (b) lidocaine-caffeine, and (c) lidocaine-urea. Blue circles: liquidus temperatures; red diamonds: solidus (eutectic) temperatures. Solid black line: NRTL-modeled liquidus; dashed black line: NRTL-modeled solidus. Blue crosses: solid–solid transition temperature for caffeine. Dashed gray lines-ideal liquidus.

Table 3 summarizes the experimental composition at the eutectic point (*x*_2,e_^exp^) and the melting temperature depressions relative to the melting points of the APIs (*T*_e_^exp^–*T*_m,1_) and the hydrotropes (*T*_e_^exp^–*T*_m,2_). It also includes the deviation of the experimentally determined eutectic temperature from those calculated if the systems behave as an ideal mixture (*T*_e_^exp^–*T*_e_^ideal^), which reflects the deviations from ideal solution behavior (γ_*i*_^L^ = 1).

**Table 3:** API-Hydrotrope Systems, Eutectic Point Compositions, and the Depression in Melting Temperature Relative to the Melting Points of the API (1), Hydrotrope (2), and Ideal System Eutectic Temperature

API(1)-hydrotrope(2)	*x*_2*,*e_^exp^ (−)	*x*_2*,*e_^ideal^ (−)	*T*_e_^exp^*–T*_e_^ideal^ (K)	*T*_e_^exp^ *–T*_m*,*1_ (K)	*T*_e_^exp^*–T*_m*,*2_ (K)
benzocaine -nicotinamide	0.20	0.30	3.82	–11.44	–50.47
benzocaine-caffeine	0.20	0.11	–6.30	–10.97	–157.91
procaine-nicotinamide	0.10	0.18	1.48	–4.31	–72.65
procaine-caffeine	0.10	0.05	–1.61	–3.10	–179.33
lidocaine-nicotinamide	0.10	0.20	0.78	–9.75	–70.61
lidocaine-caffeine	0.10	0.07	0.96	–2.16	–170.92

The binary mixtures of APIs with nicotinamide (Figures 3a, 4a, and 5a) and caffeine (Figures 3b, 4b, and 5b) exhibit near-ideal solution behavior, showing only minimal deviations from ideality anticipated by lnγ_1_^∞^ and lnγ_2_^∞^ values, as discussed in Section 3.1. Consequently, the composition at the eutectic point and the depression in melting temperatures are primarily influenced by the melting properties of the APIs and hydrotropes. Benzocaine, procaine, and lidocaine have lower melting temperatures compared to nicotinamide and caffeine (see Table 1), which shifts the eutectic point to higher mole fractions of the APIs and results in smaller melting point depressions at the eutectic points. The values of (*x*_2,e_^exp^) indicate that only small amounts of hydrotropes are needed to form eutectic mixtures, with mole fractions of the hydrotrope ranging from 0.1 to 0.2 at the eutectic points. In the API-nicotinamide systems (Figures 3a, 4a, and 5a), nicotinamide has higher melting enthalpy and entropy (see Tables 1) compared to caffeine, which decreases the slope of the nicotinamide solubility lines and shifts the eutectic point toward the API-rich side in the phase diagram. This results in smaller depressions in the melting temperatures at the eutectic point relative to the ideal solutions (*T*_e_^exp^–*T*_e_^ideal^). However, the depressions relative to the APIs (*T*_e_^exp^–*T*_m,1_) and hydrotrope (*T*_e_^exp^–*T*_m,2_) are close to API-caffeine systems. In contrast, caffeine’s lower melting enthalpy and entropy (see Table 1) compared to nicotinamide, increases the steepness of its solubility lines, shifting the eutectic point toward the caffeine-rich side in the phase diagram. Additionally, caffeine’s solid–solid transition enthalpies (see Table 1) are much smaller than its melting enthalpies, causing the slopes of the caffeine liquidus lines to become slightly less steep below the solid–solid transition temperatures. This effect is less pronounced in the lidocaine-caffeine system due to lidocaine’s lower melting enthalpy and entropy compared to benzocaine and procaine (see Table 1). The reduction in lidocaine’s melting properties increases the slope of the lidocaine solubility lines and shifts the eutectic point further toward the API-rich side in the phase diagram (see Figure 5b), resulting in smaller (*T*_e_^exp^–*T*_e_^ideal^) values. In the API-urea systems (Figures 3c, 4c, and 5c), APIs are not fully miscible in the liquid phase, resulting in the coexistence of SLE and LLE in the API-urea binary mixtures. This behavior can be attributed to urea’s acyclic and nonaromatic molecular structure, which leads to unfavored interactions with APIs while exhibiting stronger self-association, resulting in the formation of self-associated clusters that can lead to liquid–liquid phase separation (LLE).

### 3.3 Modeling the SLE Phase Diagram of the Ternary Systems

The ternary API-hydrotrope-water SLE phase diagrams were modeled at 310.15 K (human body temperature) using eq 2 and the NRTL model, with the binary interaction parameters presented in Table 2 and the compounds melting properties in Table 1. The ternary SLE diagrams for the API-hydrotrope-water systems of benzocaine, procaine, and lidocaine are shown in Figures 6, 7, and 8, respectively. The studied temperature of 310.15 K is lower than the melting temperatures of the pure hydrotropes (see Table 1), indicating that the pure hydrotropes are solid at the solution temperature. The phase diagrams show that the API-hydrotrope-water systems at the studied temperature are eutectic systems. On the right side of the figures, a zoomed-in view of the phase diagrams is provided for a better visualization of the solubility lines and eutectic points. Since, the region for the APIs-urea systems is too small to be visible and is not shown.

**Figure 6:**
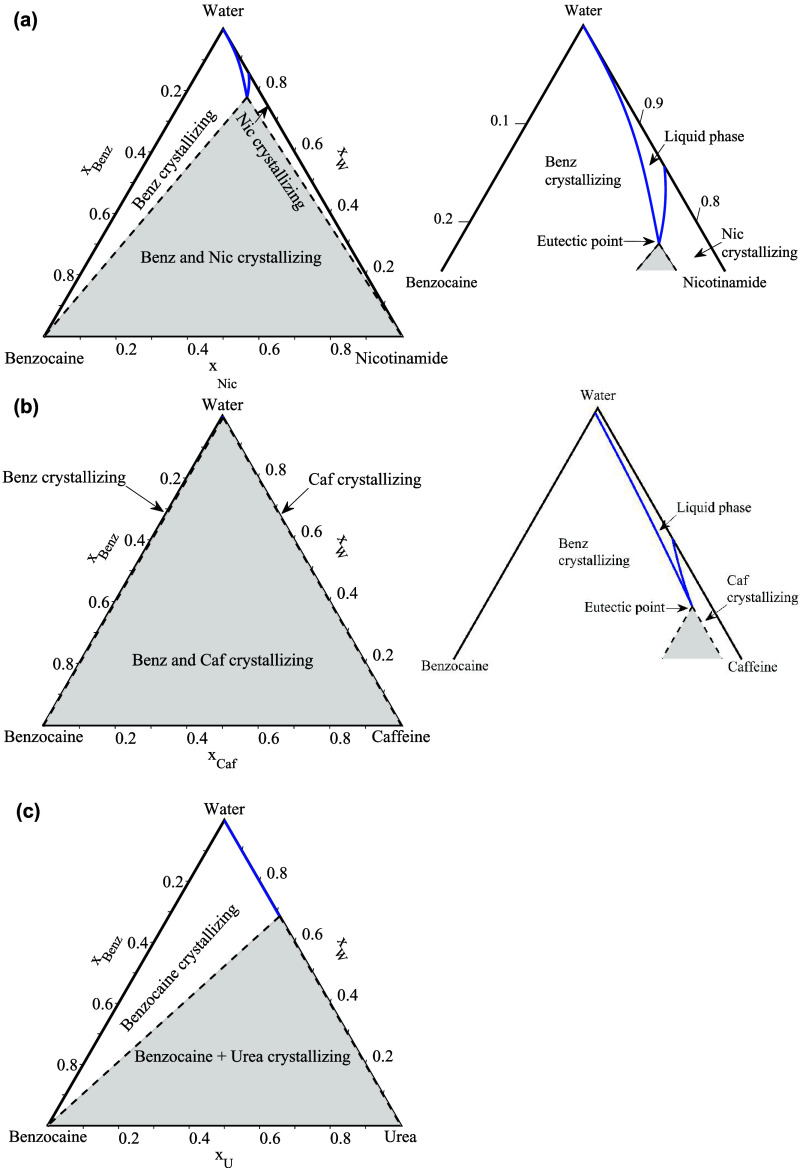
Calculated SLE phase diagrams of the benzocaine-hydrotrope-water systems at 310.15 K with the NRTL model. (a) Benzocaine-nicotinamide-water, (b) benzocaine-caffeine-water, and (c) benzocaine-urea-water. The right side of each figure shows a zoomed-in view except for the urea system.

**Figure 7:**
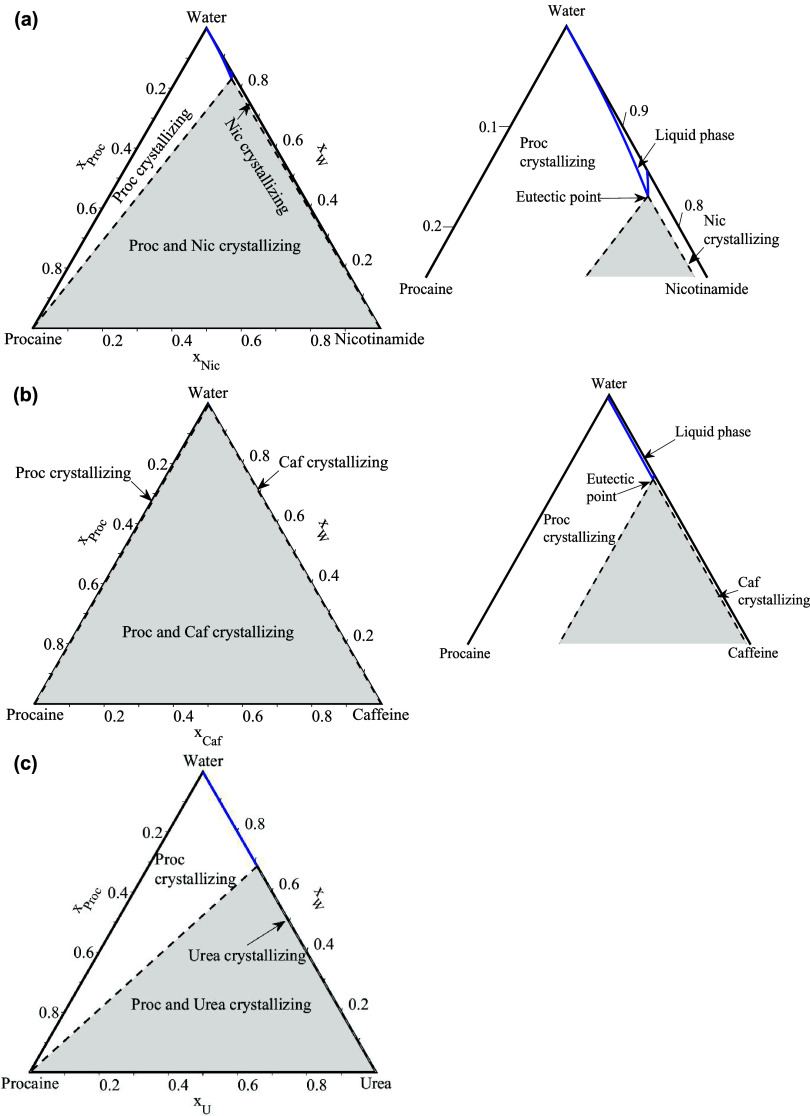
Calculated SLE phase diagrams of the procaine-hydrotrope-water systems at 310.15 K with the NRTL model. (a) Procaine-nicotinamide-water, (b) procaine-caffeine-water, and (c) procaine-urea-water. The right side of each figure shows a zoomed-in view, except for the urea system.

**Figure 8:**
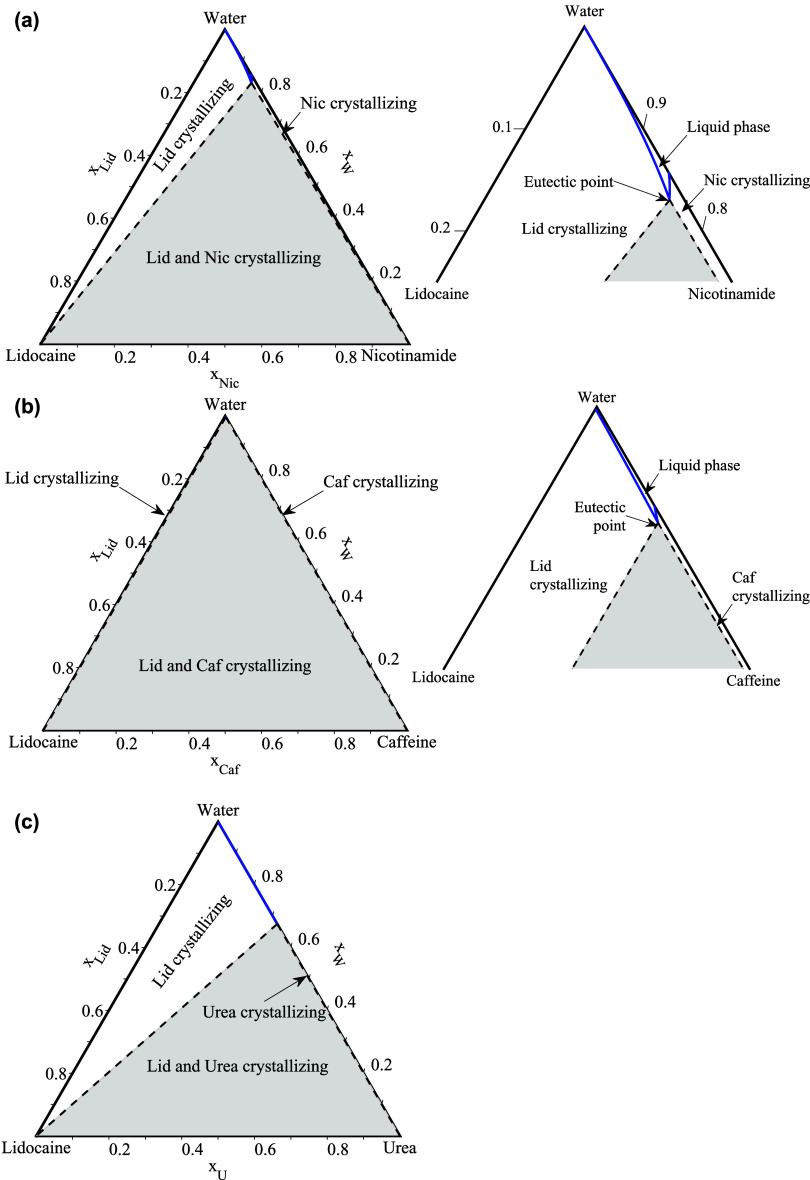
Calculated SLE phase diagrams of the lidocaine-hydrotrope-water systems at 310.15 K with the NRTL model. (a) lidocaine-nicotinamide-water, (b) lidocaine-caffeine-water, and (c) lidocaine-urea-water. The right side of each figure shows a zoomed-in view, except for the urea system.

In Figures 6, 7, and 8, the blue lines represent the solubility isotherms of the system at 310.15 K. Each isotherm consists of two solubility lines: the API solubility line and the hydrotrope solubility line, which intersect at the eutectic point. At this point, the API solubility enhancement in the ternary API-hydrotrope-water systems is the highest.^38^ The shape of the API and hydrotrope solubility lines varies depending on the melting properties of the selected APIs and hydrotropes and their activity coefficients in the liquid phase, which influence the position of the eutectic point and the extent of API solubility enhancement in the ternary API-hydrotrope-water solution. In cases where the eutectic point is located toward the lower left of the phase diagram (closer to the API side), it increases the API mole fraction in the liquid solution and maximizes the solubility enhancement of the API, as demonstrated in our detailed theoretical study.^38^

For benzocaine, the analysis in Sections 3.1 and 3.2.3 revealed that among the three selected hydrotropes, benzocaine interacts more favorably with nicotinamide and caffeine (strong API-hydrotrope interactions) than with urea. Additionally, as previously discussed in Sections 3.1 and 3.2.2, nicotinamide exhibits moderate interactions with water relative to urea and caffeine (moderate hydrotrope-water interactions). As a result, in the benzocaine–nicotinamide–water system, the eutectic point is located closer to the API (benzocaine)-rich region (on the left side of Figure 6a), indicating that the benzocaine mole fraction increases along the API solubility line with the addition of nicotinamide, reaching its maximum at the eutectic point.

In the benzocaine-caffeine-water system (Figure 6b) and the benzocaine-urea-water system (Figure 6c), although the addition of these hydrotropes increases the benzocaine mole fraction along the API solubility line, the solubility enhancement remains less significant compared to the benzocaine–nicotinamide–water system. This is due to the relatively less favored interactions between caffeine and water (compared to nicotinamide and water) and benzocaine and urea (compared to benzocaine and nicotinamide), as shown previously in Table 2, resulting in a lower overall solubility enhancement effect. Similar observations were made for the procaine and lidocaine systems, as shown in Figures 7 and 8.

In Table 4, the API composition (*x*_e,API_), hydrotrope composition (*x*_e,hydrotrope_), and solubility enhancement factor (Φ) at the eutectic point in the ternary API-hydrotrope-water systems at 310.15 K, calculated using the NRTL model are shown. The systems with nicotinamide show the highest values of the API mole fractions (*x*_e,API_) and the API solubility enhancement factors Φ at the eutectic point in the ternary systems. The API solubility enhancement factors Φ in the urea and caffeine systems are almost in the same range and much lower compared to the nicotinamide-based ternary systems.

**Table 4:** API Composition (*x*_e,API_), Hydrotrope Composition (*x*_e,hydrotrope_), and Solubility Enhancement Factor (Φ) at the Eutectic Point in the Ternary API-Hydrotrope-Water Systems at 310.15 K, Calculated Using the NRTL Model^a^

API-hydrotrope-water system	*x*_e,API_	*x*_e,hydrotrope_	*T*_e,exp_	Φ
benzocaine-nicotinamide-water	4.3923 × 10^–2^	0.1774	308.95	377.00
benzocaine-caffeine-water	3.9947 × 10^–4^	0.0044	308.80	3.65
benzocaine-urea-water	7.0401 × 10^–4^	0.3141	310.85	9.30
procaine-nicotinamide-water	1.2095 × 10^–2^	0.1571	310.60	132.40
procaine-caffeine-water	1.5361 × 10^–4^	0.0014	308.60	2.20
procaine-urea-water	1.5844 × 10^–4^	0.3138	310.85	2.10
lidocaine-nicotinamide-water	1.2606 × 10^–2^	0.1574	311.35	221.50
lidocaine-caffeine-water	1.7714 × 10^–4^	0.0035	311.05	2.60
lidocaine-urea-water	7.4000 × 10^–5^	0.3200	311.50	1.60

a The experimental melting temperature (*T*_e,exp_) measured at the eutectic point is included to validate the modeling results.

The modeling results of the ternary API-hydrotrope-water systems are in agreement with the theoretical findings of our previous theoretical study.^38^ Namely, the maximum API solubility is achieved at the eutectic point in the ternary eutectic system, which is determined by the combined effect of binary interactions among the API, hydrotrope, and water and their melting properties. The optimal hydrotrope should exhibit strong interactions with the API to enhance solubility while also interacting favorably with water. However, the hydrotrope-water interactions should not exceed the API-hydrotrope interactions, as excessively strong hydrotrope-water interactions can reduce the solubility enhancement effect. In Figure S24a–i in the Supporting Information file, a common alternative representation for the ternary phase diagrams for each of the nine studied systems at 310.15 K is shown.

### 3.4 Validation of NRTL Model Predictions for Ternary API-Hydrotrope-Water Systems

To validate the predicted ternary phase diagrams of API-hydrotrope-water systems for each of the nine systems, three specific system compositions were selected from the NRTL modeled isotherm at 310.15 K: one at the eutectic point, one at the API solubility line, and one at the hydrotrope solubility line. The API-hydrotrope-water mixtures corresponding to these compositions were prepared following the method described in Section 2.4, and their melting temperatures were measured using the temperature variant method. In Table 4, the experimentally measured melting temperatures (*T*_e,exp_) at the eutectic composition in the ternary API-hydrotrope-water systems are shown. In Figure 9, the obtained experimental melting temperatures at the eutectic point (*T*_e,exp_), the API solubility line, and the hydrotrope solubility line are presented. The experimentally determined melting temperatures for the three selected compositions for all nine systems deviate from the isotherm temperature (310.15 K) by less than 1%. This strong agreement suggests that the binary interaction parameters, which were obtained by fitting the experimental SLE data of the three binary systems: API-hydrotrope, API-water, and hydrotrope-water, accurately predict the SLE of the ternary API-hydrotrope-water system.

**Figure 9:**
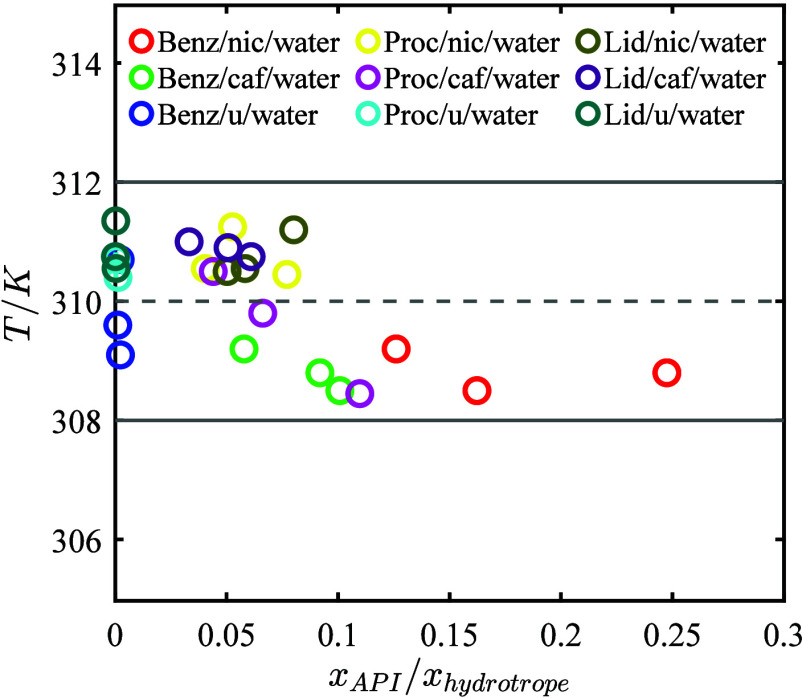
Validation of the NRTL model predictions for the ternary API-hydrotrope-water systems at 310.15 K. Colored markers represent different API-hydrotrope-water systems, with three selected compositions for each system (at the eutectic point, one at the API solubility line, and one at the hydrotrope solubility line). The dashed blue line: the predicted temperature of 310.15 K. The solid blue lines: the temperature range of 310.15 ± 2 K.

### 3.5 Applications of the Ternary SLE Phase Diagrams

This section demonstrates how ternary API–hydrotrope–water SLE phase diagrams can be applied to guide the selection of an optimal API–hydrotrope molar ratio and the required amount of water to prepare a solution with a targeted API solubility, while ensuring that the drug solution remains in the liquid state at the intended storage or administration temperature.

In Figure 10a, it is shown how the ternary API-hydrotrope-water phase diagram, using the benzocaine-nicotinamide–water system as an example, aids in selecting the initial API-hydrotrope molar composition and determining the necessary amount of water to obtain a liquid solution with maximum API solubility at a specific temperature. First, a line (the green line) starting from the top corner of the ternary phase diagram, passing through the eutectic point of the ternary system is drawn. The intersection of this line with the base of the ternary diagram at *x*_e,water_ = 0, represents the initial composition of the binary API–hydrotrope mixture required to prepare a solution with the maximum API solubility at the eutectic point. For the regarded system, starting with the binary benzocaine-nicotinamide mixture with molar composition *x*_API_ = 0.2 and *x*_hydrotrope_ = 0.8, the water is added along the green stoichiometric line (dilution path) up to the eutectic point in the ternary mixture (maximum solubility point at 310.15 K). However, following the conventional approach of using the binary eutectic composition (*x*_API_ = 0.8 and *x*_hydrotrope_ = 0.2) as a starting point and adding water (along the red line) leads to only a small solubility enhancement of the API and does not yield the highest possible API concentration in an aqueous solution.

**Figure 10:**
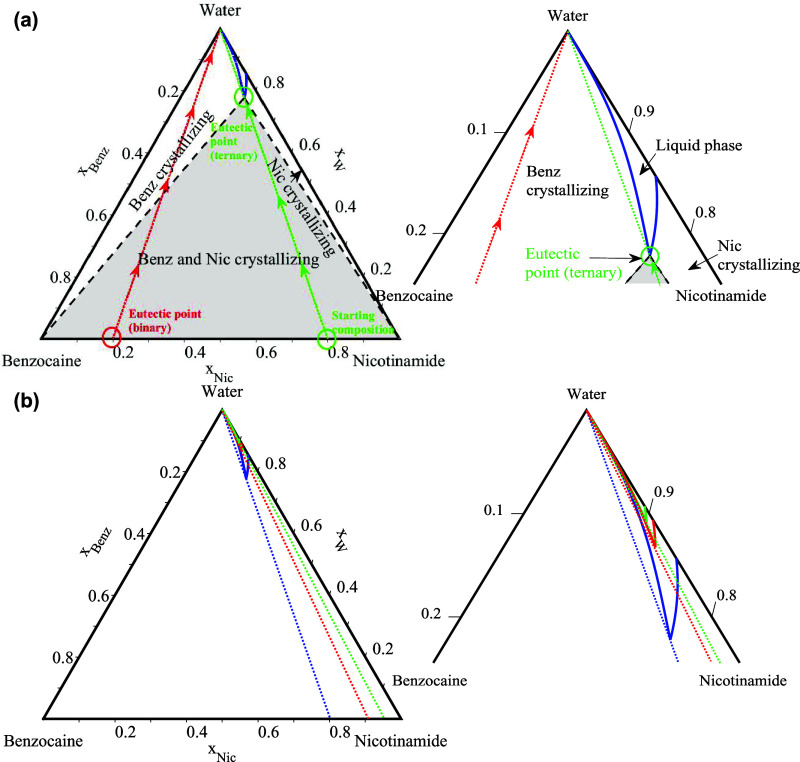
Calculated SLE phase diagrams of the benzocaine-nicotinamide-water system: (a) at 310.15 K with the NRTL model. The red and green lines represent the stoichiometric lines. (b) At 310.15 K (blue line), 298.15 K (red line), and 293.15 K (green line) with the NRTL model. The right side of each figure shows a zoomed-in view.

When preparing the API-hydrotrope-water solutions, it is crucial not only to maximize API solubility but also to ensure formulation stability under temperature fluctuations. The NRTL model can be used to predict solubility isotherms at different temperatures, as shown for the benzocaine-nicotinamide-water systems in Figure 10b (310.15 K, blue; 298.15 K, red; and 293.15 K, green). The SLE data at varying temperatures aids the selection of the correct compositions of the components in the solution that prevents crystallization of the API or hydrotrope in the final product during storage, transport, or use. This ensures that both the API and hydrotrope remain in the liquid solution, preventing crystallization and maintaining stability during storage and administration. For example, if the storage temperature is 293.15 K (see Figure 10b, green isotherm), the solution should be prepared at the eutectic point of this isotherm to ensure it remains liquid at this temperature.

## 4 Conclusions

This study aims to analyze and understand hydrotropy from a thermodynamic perspective by integrating experimental SLE data with thermodynamic modeling. It proposes a thermodynamic-based approach for predicting the SLE phase diagram of a ternary API-excipient-water system, with a focus on hydrotropes that are solid at the solution temperature as pharmaceutical excipients to enhance API solubility in water. The approach considers the solvent effect, accounting for the interactions between the API and hydrotrope, API and water and, hydrotrope and water. First, the SLE data of API-hydrotrope, API-water, and hydrotrope-water binary systems at different temperatures were experimentally measured using high-throughput methods. Second, the binary interaction parameters of each sub-binary system were obtained from the experimental data using the NRTL model. Then, the SLE phase diagram of a ternary API-hydrotrope-water system was predicted using the melting properties of the components and their activity coefficients in the ternary liquid solution. The activity coefficients were calculated using the NRTL model, and the previously obtained binary interaction parameters. The approach was applied to nine ternary systems, consisting of three APIs (benzocaine, lidocaine, and procaine) and three hydrotropes (nicotinamide, caffeine, and urea) with water as solvent. The NRTL-modeled solubility of the API at 310.15 K in the ternary solutions showed strong agreement with the experimental data, validating the proposed approach.

The findings demonstrate that nicotinamide and caffeine form eutectic mixtures with APIs. Binary API-hydrotrope phase diagrams alone are inadequate for selecting the optimal excipient, as they do not account for API-water and hydrotrope-water interactions. Ternary SLE phase diagrams incorporate the solvent effect, offering a more accurate basis for excipient selection. The highest API solubility in the ternary API-hydrotrope-water mixture was found at the eutectic point of the ternary system beyond which the addition of more API or hydrotrope leads to precipitation. Additionally, the ternary SLE phase diagrams aid in identifying the optimal starting compositions for API-hydrotrope mixtures and the required water amount to achieve maximum API solubility (eutectic point) while preventing the crystallization of APIs or hydrotropes at relevant storage and application temperatures. The study emphasizes that favorable API-hydrotrope and hydrotrope-water interactions are crucial for API solubility enhancement, with nicotinamide showing the highest solubilization efficiency among the studied hydrotropes.

## Supplementary Material



## Data Availability

The data supporting this article have been included in the main manuscript and the Supporting Information.

## Notes

PXRD patterns of the three APIs and three hydrotropes at ambient temperature, compared to calculated patterns; PXRD pattern of the binary mixtures of the APIs and hydrotropes (1:1 molar ratio), compared to pure APIs and hydrotropes at ambient temperature; PXRD patterns of benzocaine (as purchased, after 20 min of grinding and the three individual polymorphs—polymorph I; polymorph II; polymorph III); Pawley profile of the of the purchased benzocaine before grinding; Pawley profile for the purchased benzocaine after 20 min of grinding; DSC curves of pure APIs, pure hydrotropes, and the API-hydrotrope binary mixtures at the eutectic points; melting temperature (*T*_m_) and enthalpy (Δ*h*_m_) of the selected APIs and hydrotropes, measured in this work and compared to literature values; solid–liquid equilibrium (SLE) data for the binary API-hydrotrope eutectic systems; measured solubility data of the model APIs and selected hydrotropes in water, respectively, using the temperature-variant method; SLE phase diagram of the urea–water system modeled using the NRTL equation and compared to experimental data; solubility of APIs in water at different temperatures calculated by the NRTL model and assuming ideal solution behavior; solubility of the hydrotropes in water, experimentally determined in this work, modeled using the NRTL equation and compared to literature data; and common alternative representation for the ternary phase diagrams for each of the nine studied systems at 310.15 K (PDF)
