# Supplementary material for: Elucidating the Solubility Enhancement of Active Pharmaceutical Ingredients through Hydrotropy: A Case of Local Anesthetics
Source: Mol Pharm. 2025 Jul 4;22(8):4953–68. doi: 10.1021/acs.molpharmaceut.5c00628 (PMC12326365; doi:10.1021/acs.molpharmaceut.5c00628)
Supplement: Supplementary file 1 [file mp5c00628_si_001.pdf]

Elucidating the Solubility Enhancement of Active Pharmaceutical Ingredients through Hydrotropy: A Case of Local Anesthetics

Sahar Nasrallah[1], Alexander Wendler[1], Sebastian A. Hallweger[2], Gregor Kieslich[2] and Mirjana Minceva[1,*]

[1] Biothermodynamics, TUM School of Life Sciences, Technical University of Munich, Maximus-von Imhof-Forum 2, Freising 85354, Germany

[2] Department of Chemistry, TUM School of Natural Sciences, Technical University of Munich, Lichtenbergstraße 4, Garching 85748, Germany

*Corresponding author e-mail: mirjana.minceva@tum.de

Powder X-ray diffraction

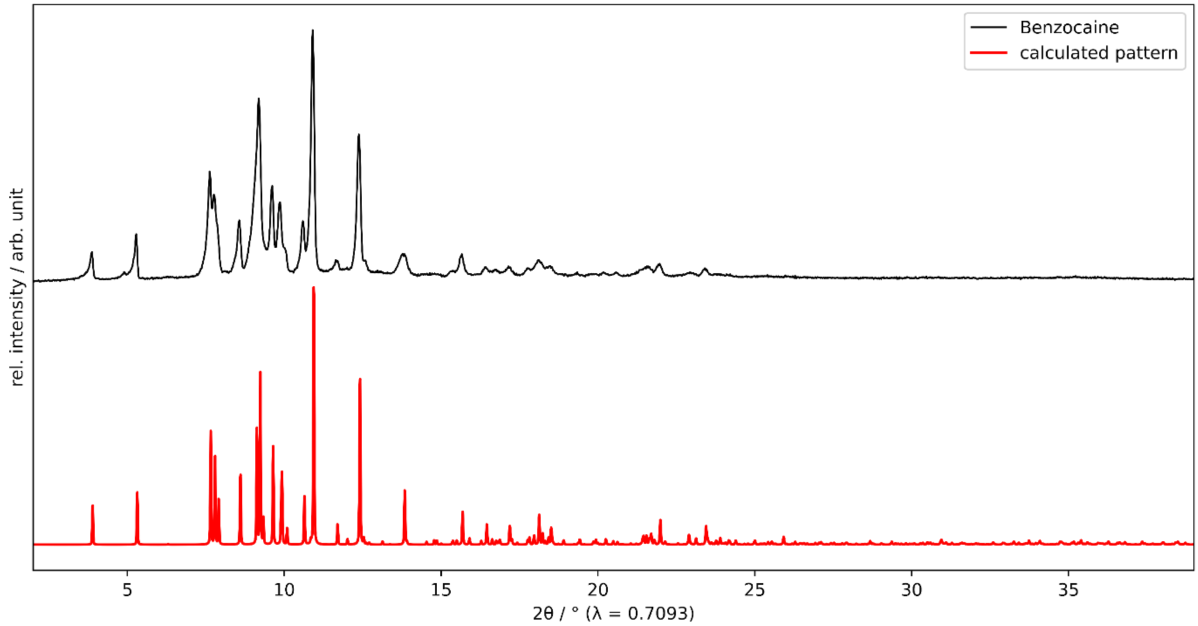

Figure S1. The PXRD pattern of the pure benzocaine sample (black) at ambient temperature compared to the calculated pattern from single-crystal X-ray diffraction (red) at 295K[1].

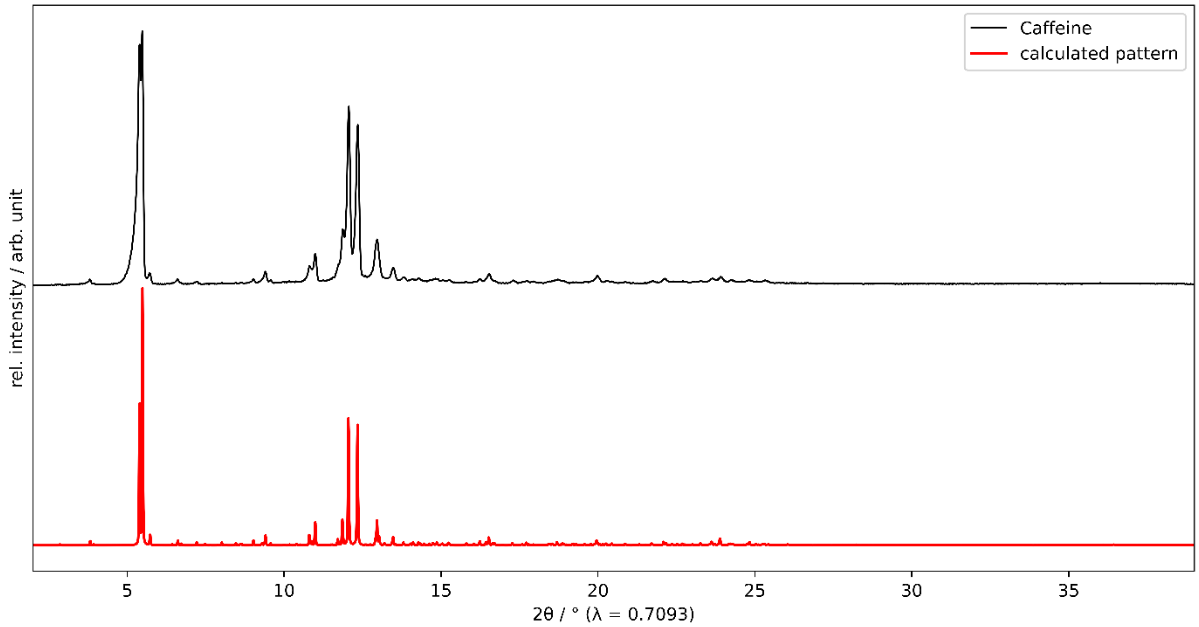

Figure S2. The PXRD pattern of the pure caffeine sample (black) at ambient temperature compared to the calculated pattern from single-crystal X-ray diffraction (red) at 295K[2].

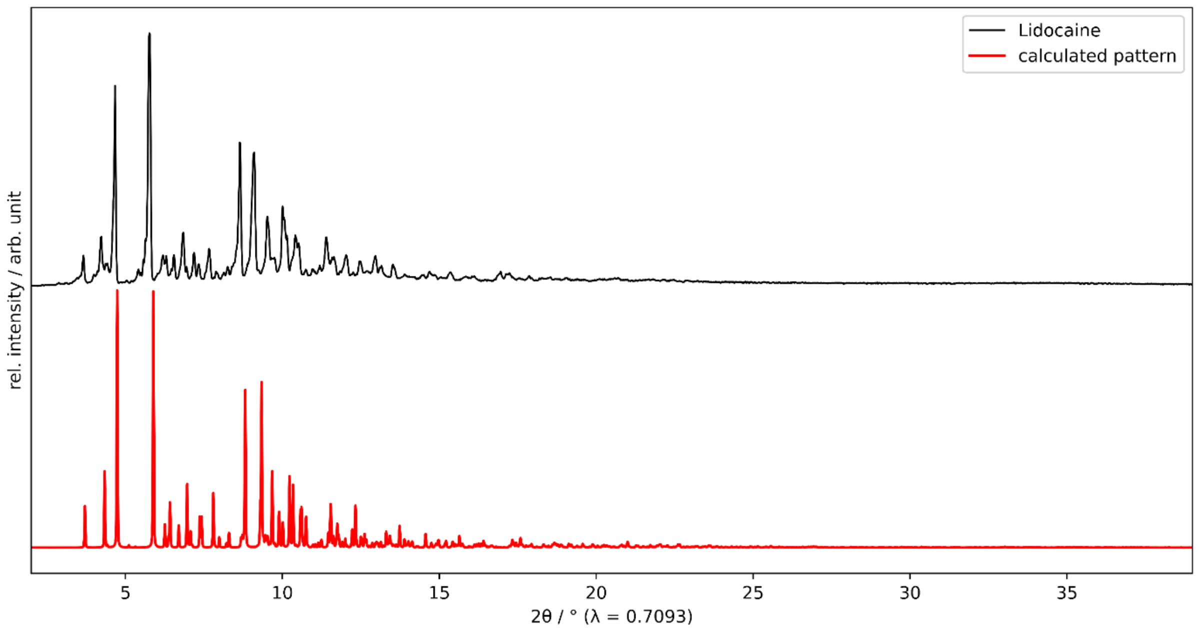

Figure S3. The PXRD pattern of the pure lidocaine sample (black) at ambient temperature compared to the calculated pattern from single-crystal X-ray diffraction (red) at 173K[3].

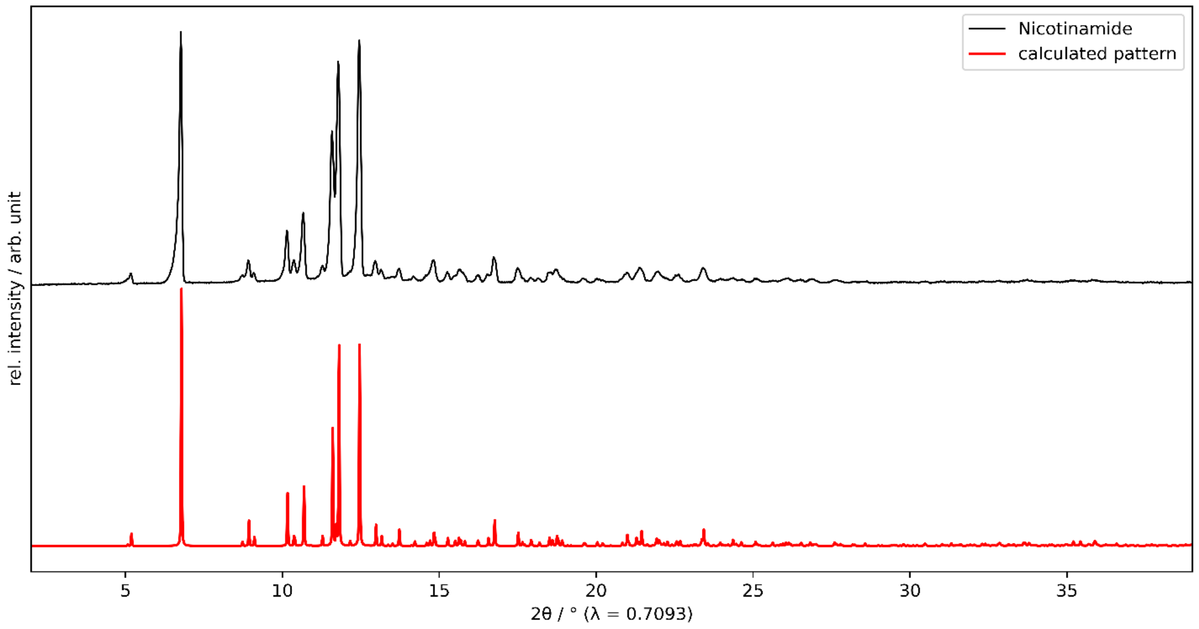

Figure S4. The PXRD pattern of the pure nicotinamide sample (black) at ambient temperature compared to the calculated pattern from single-crystal X-ray diffraction (red) at 295K[4].

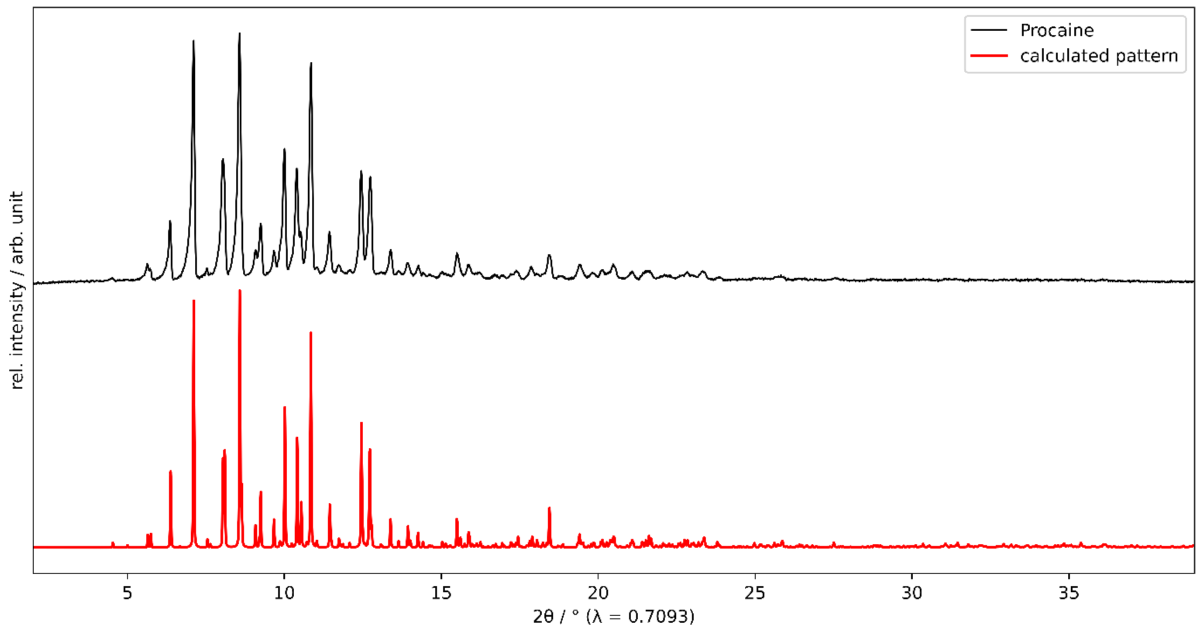

Figure S5. The PXRD pattern of the pure procaine sample (black) at ambient temperature compared to the calculated pattern from single-crystal X-ray diffraction (red) at 295K[5].

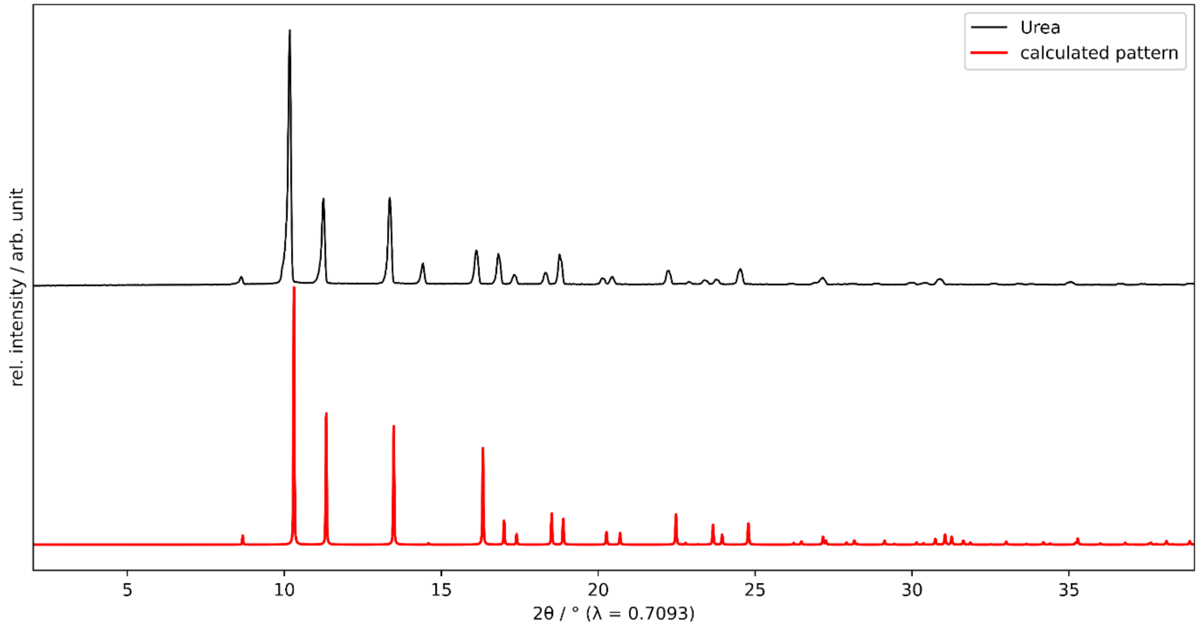

Figure S6. The PXRD pattern of the pure urea sample (black) at ambient temperature compared to the calculated pattern from neutron diffraction (red) at 123K[6].

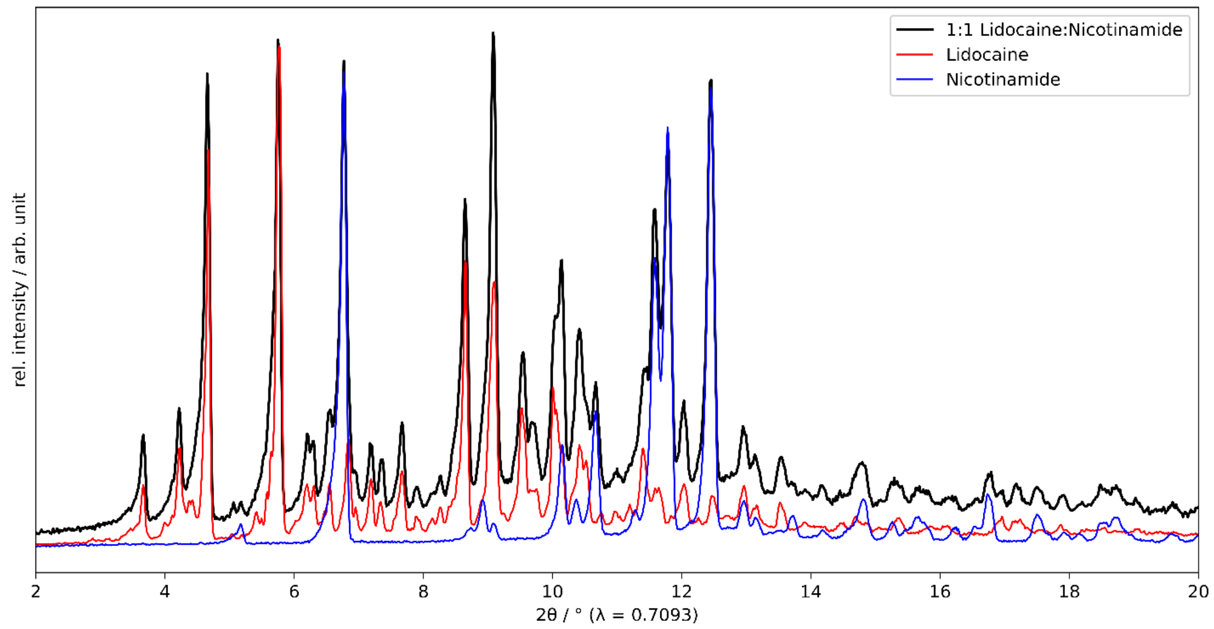

Figure S7. The PXRD pattern of the binary mixture of lidocaine and nicotinamide (black) compared to pure lidocaine (red) and nicotinamide (blue) at ambient temperature.

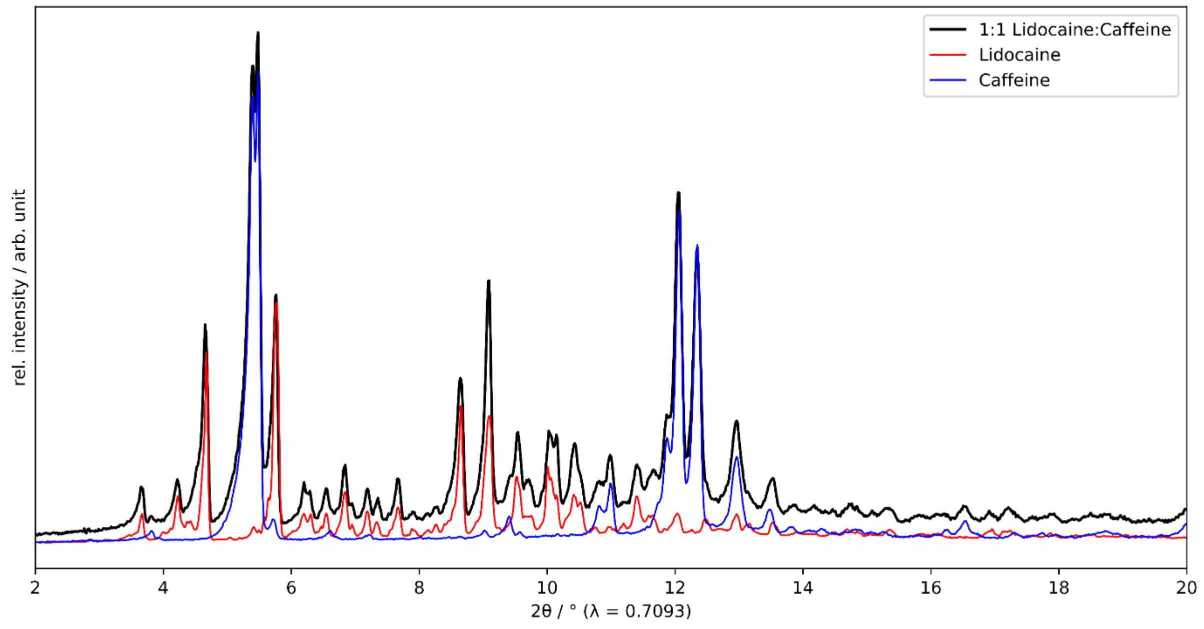

Figure S8. The PXRD pattern of the binary mixture of lidocaine and caffeine (black) compared to pure lidocaine (red) and caffeine (blue) at ambient temperature.

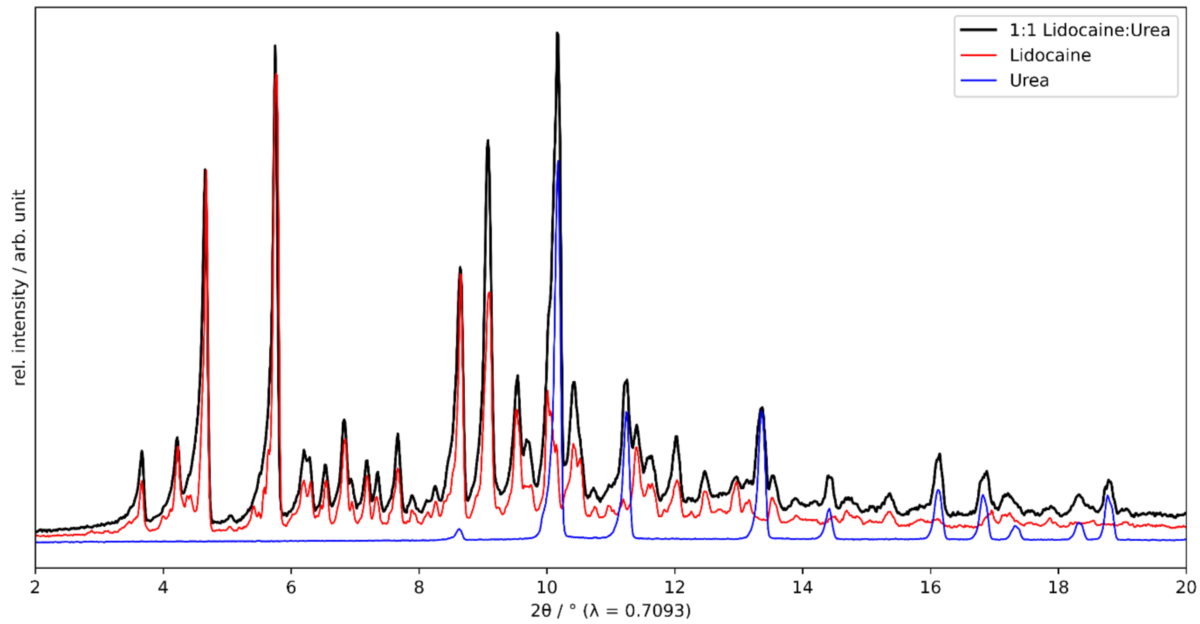

Figure S9. The PXRD pattern of the binary mixture of lidocaine and urea (black) compared to pure lidocaine (red) and urea (blue) at ambient temperature.

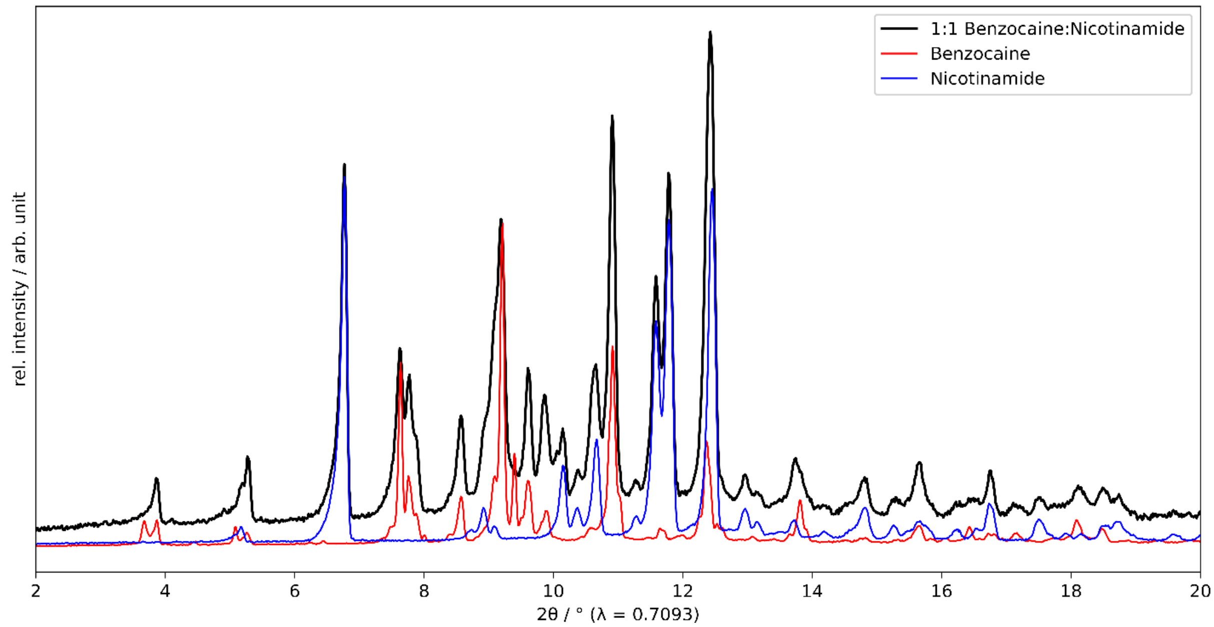

Figure S10. The PXRD pattern of the binary mixture of benzocaine and nicotinamide (black) compared to pure benzocaine (red) and nicotinamide (blue) at ambient temperature.

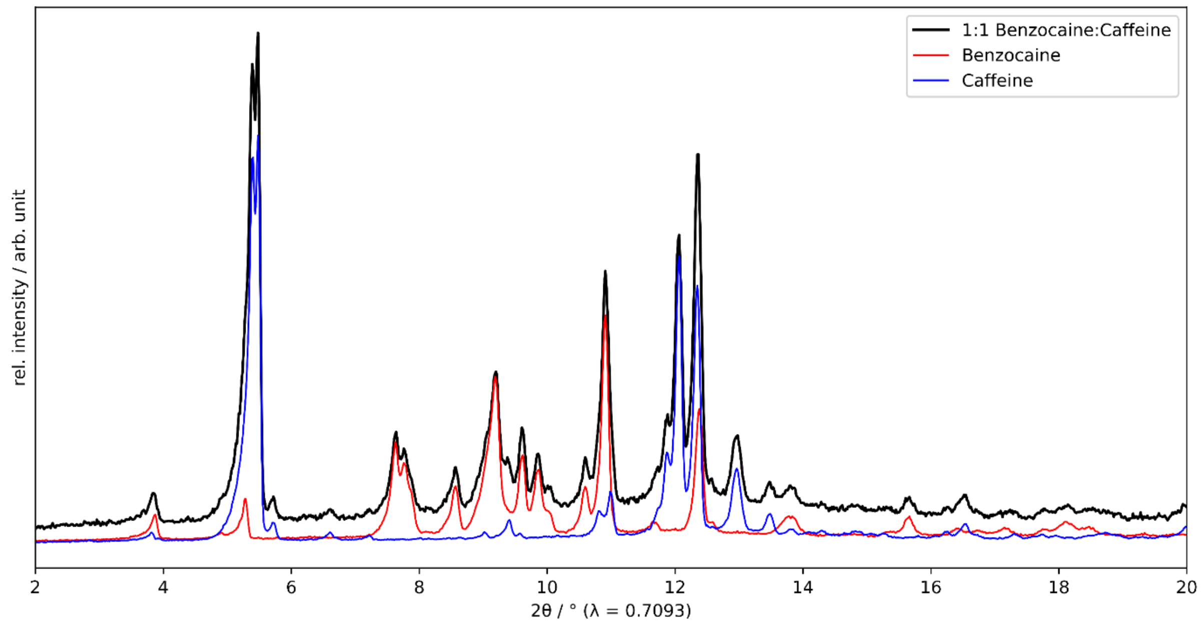

Figure S11. The PXRD pattern of the binary mixture of benzocaine and caffeine (black) compared to pure benzocaine (red) and caffeine (blue) at ambient temperature.

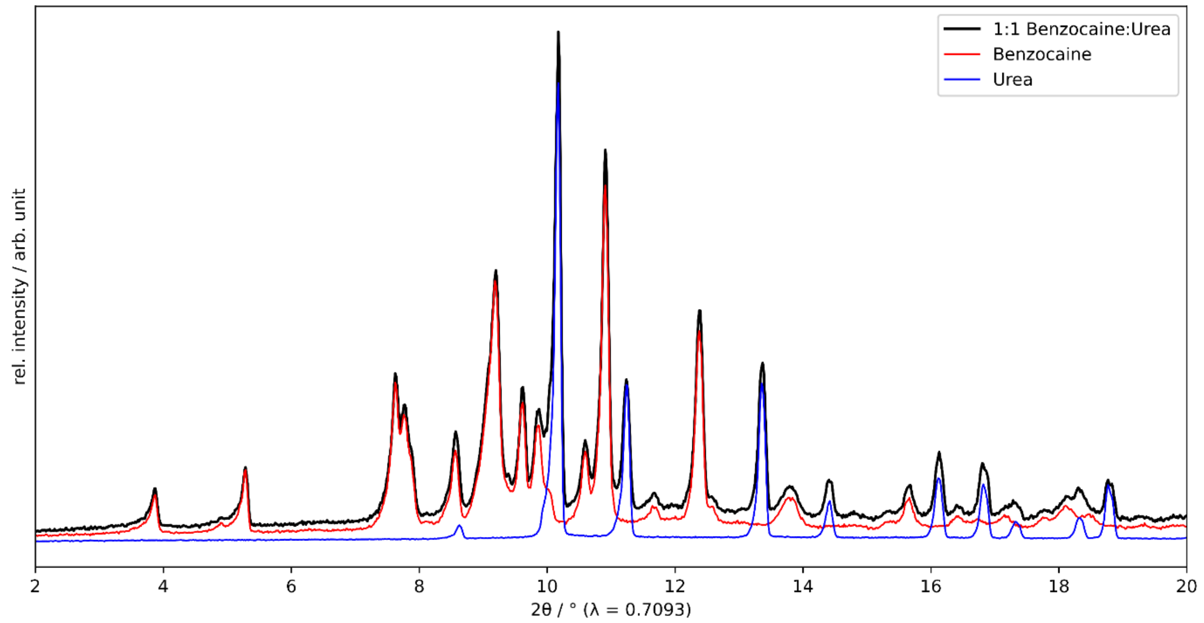

Figure S12. The PXRD pattern of the binary mixture of benzocaine and urea (black) compared to pure benzocaine (red) and urea (blue) at ambient temperature.

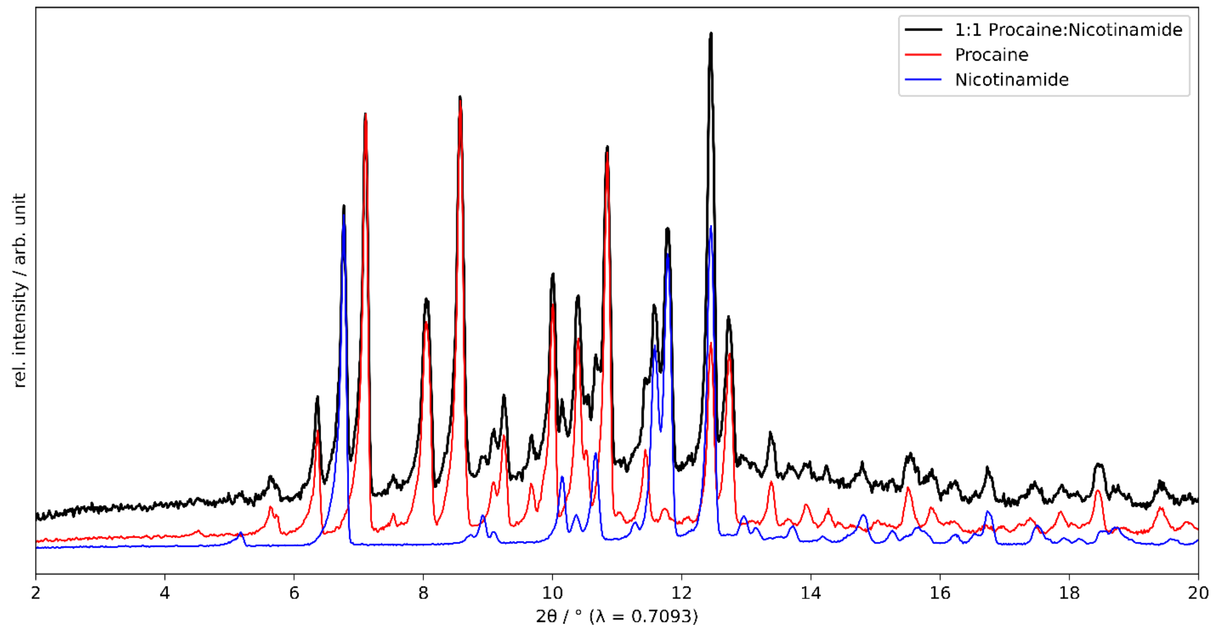

Figure S13. The PXRD pattern of the binary mixture of procaine and nicotinamide (black) compared to pure procaine (red) and nicotinamide (blue) at ambient temperature.

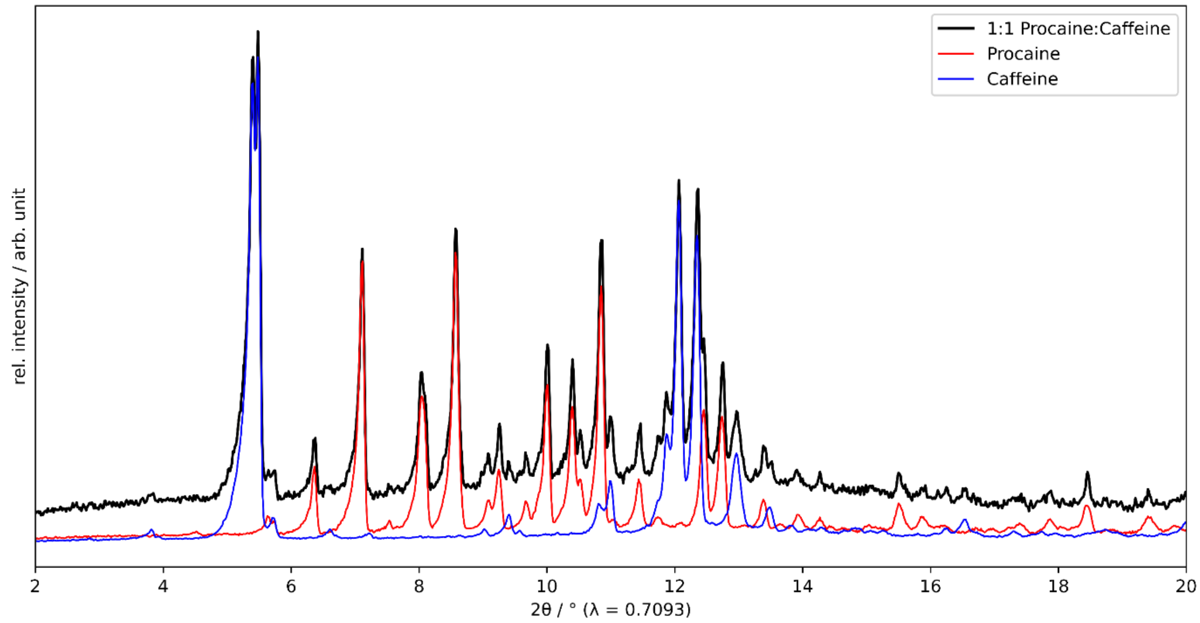

Figure S14. The PXRD pattern of the binary mixture of procaine and caffeine (black) compared to pure procaine (red) and caffeine (blue) at ambient temperature.

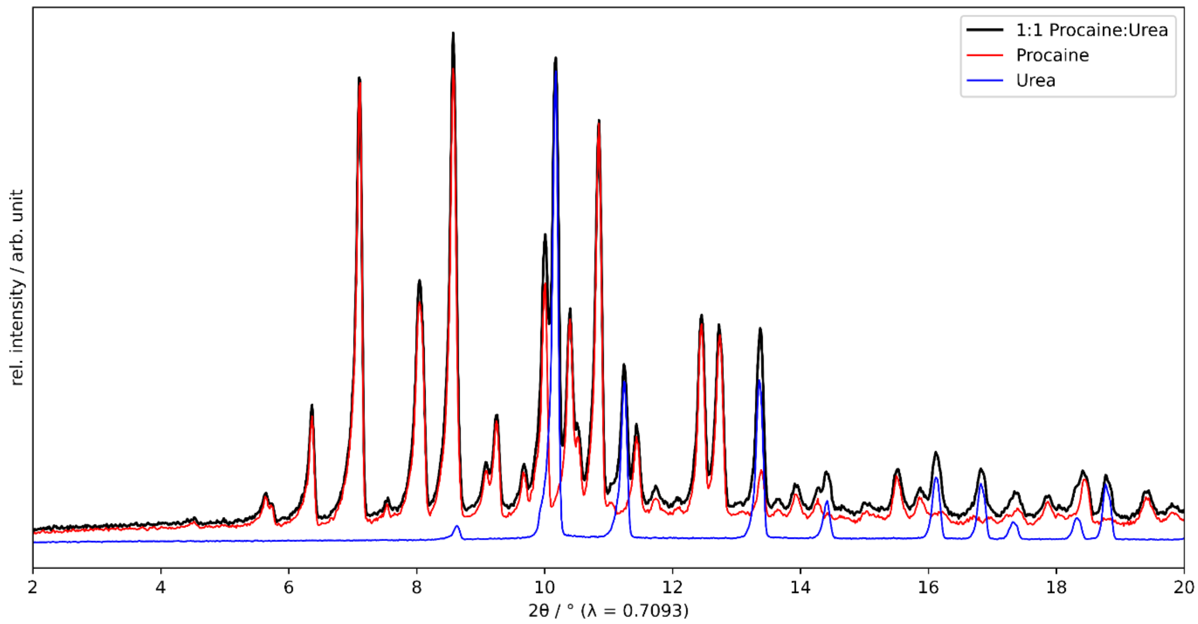

Figure S15. The PXRD pattern of the binary mixture of procaine and urea (black) compared to pure procaine (red) and urea (blue) at ambient temperature.

Phase analysis benzocaine

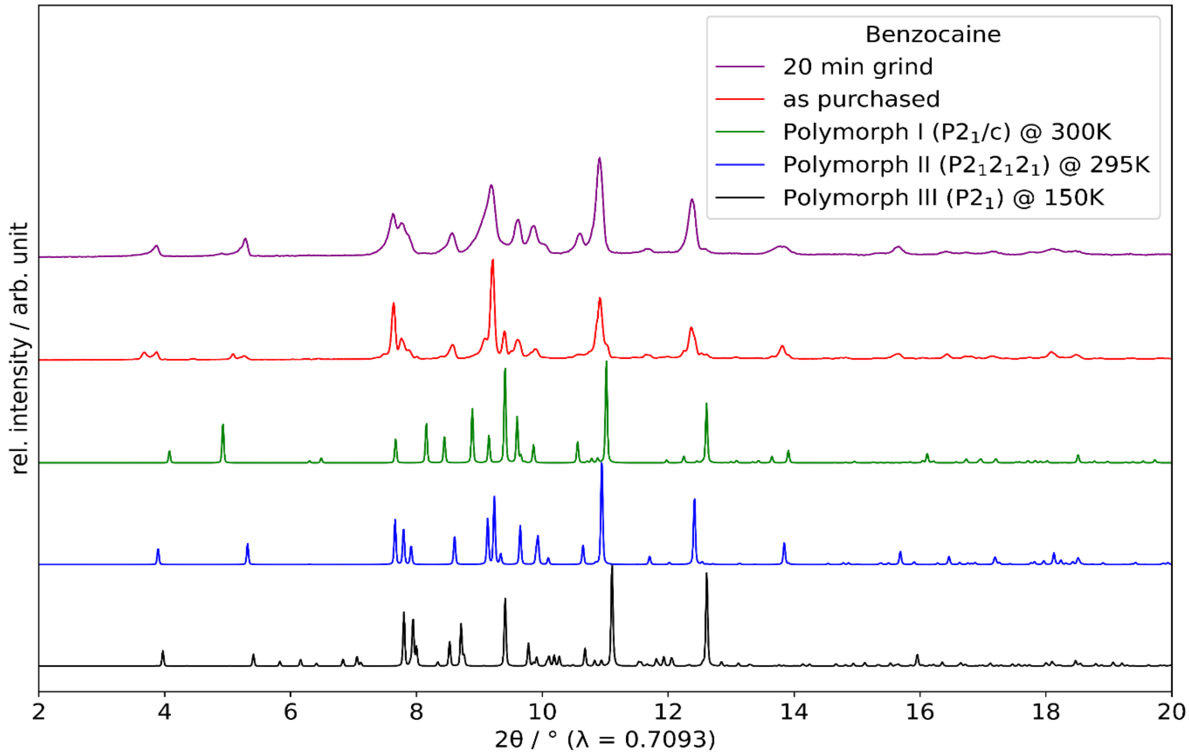

Figure S16. Stacked plot of the capillary measurements of benzocaine after 20 min grind (purple) and as purchased (red) with calculated patterns of the different polymorphs of benzocaine (polymorph I [7]: green; polymorph II [1]: blue; polymorph III [7]: black).

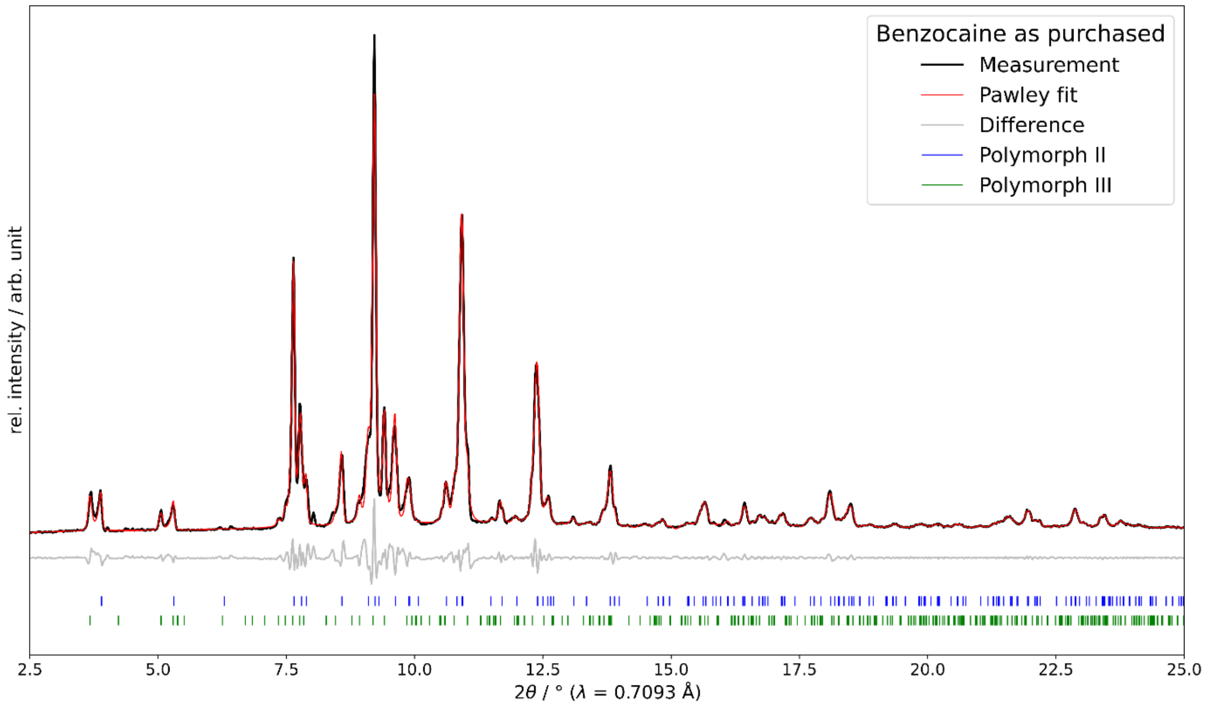

Figure S17. Pawley profile fit of benzocaine sample before grinding based on the polymorph II and III structure. Both phases were necessary to obtain a good quality fit, indicating a phase mixture of polymorph II and III before grinding.

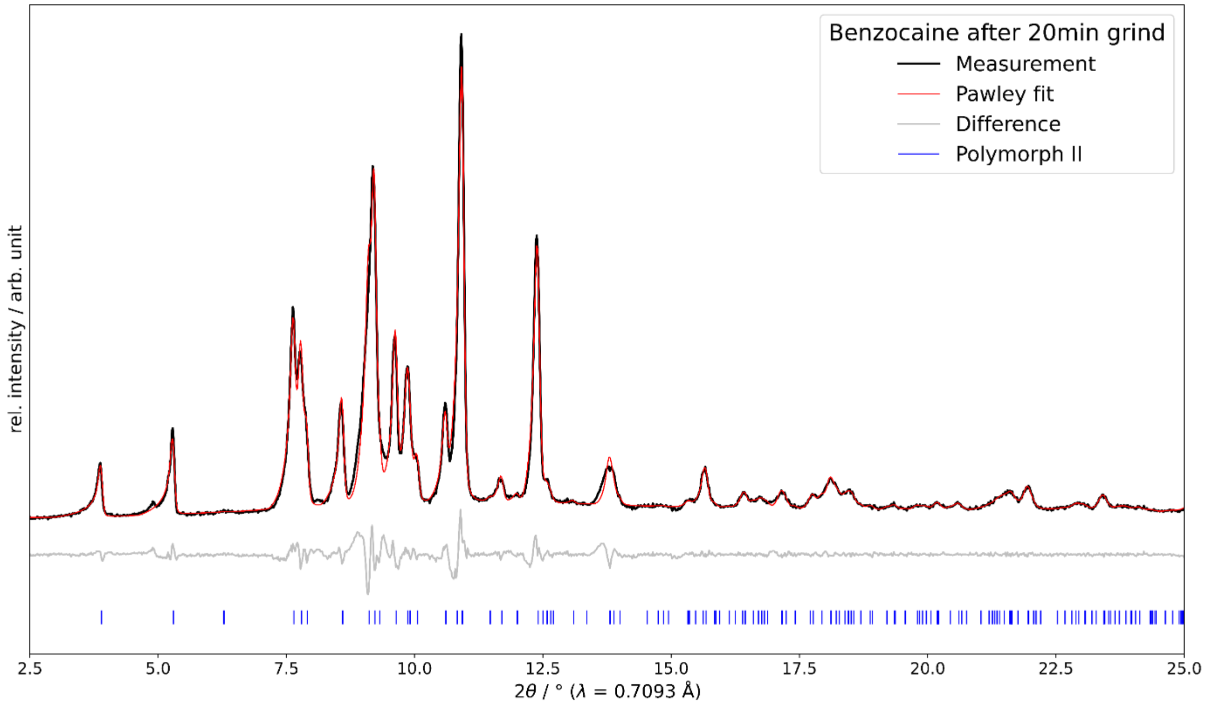

Figure S18. Pawley profile fit of the benzocaine sample after 20 min grinding based on the polymorph II structure. The quality of the Pawley fit, especially that almost all reflections (only exception is a a residual reflection of polymorph III at approximately 4.8°) can be assigned to the expected hkl ticks, indicates that the majority of the phase is polymorph II after grinding.

Differential scanning calorimetry (DSC)

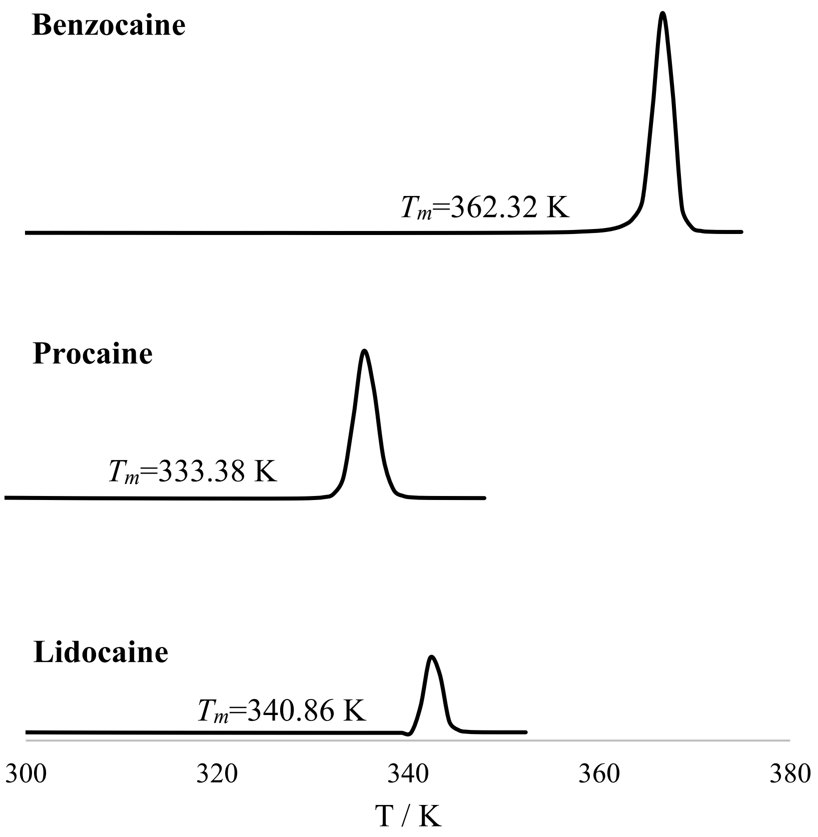

Figure S19. Differential scanning calorimetry curve of model APIs.

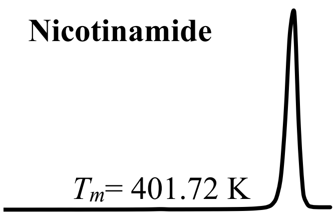

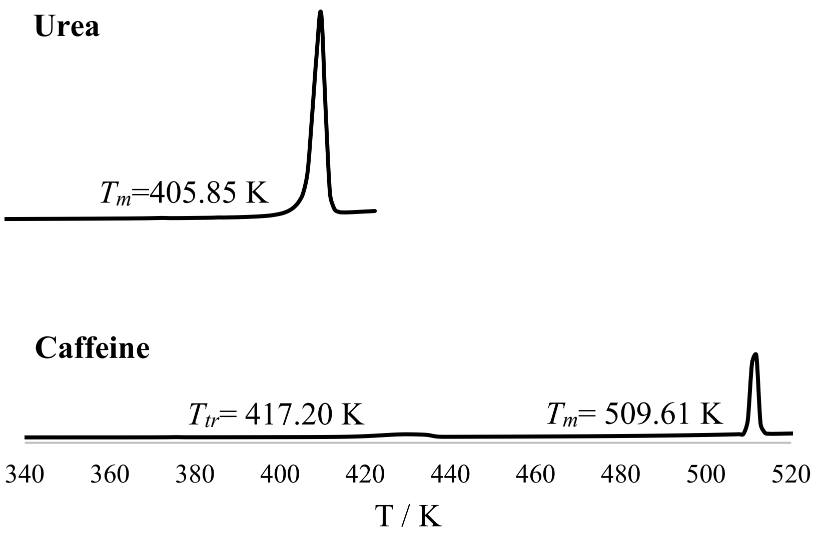

Figure S20. Differential scanning calorimetry curve of hydrotropess.

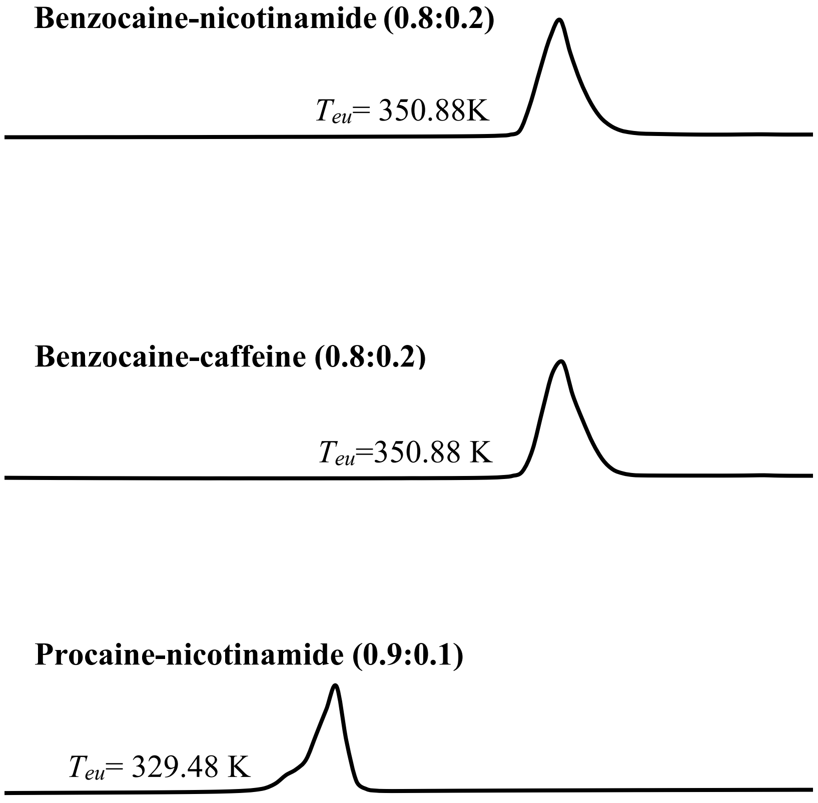

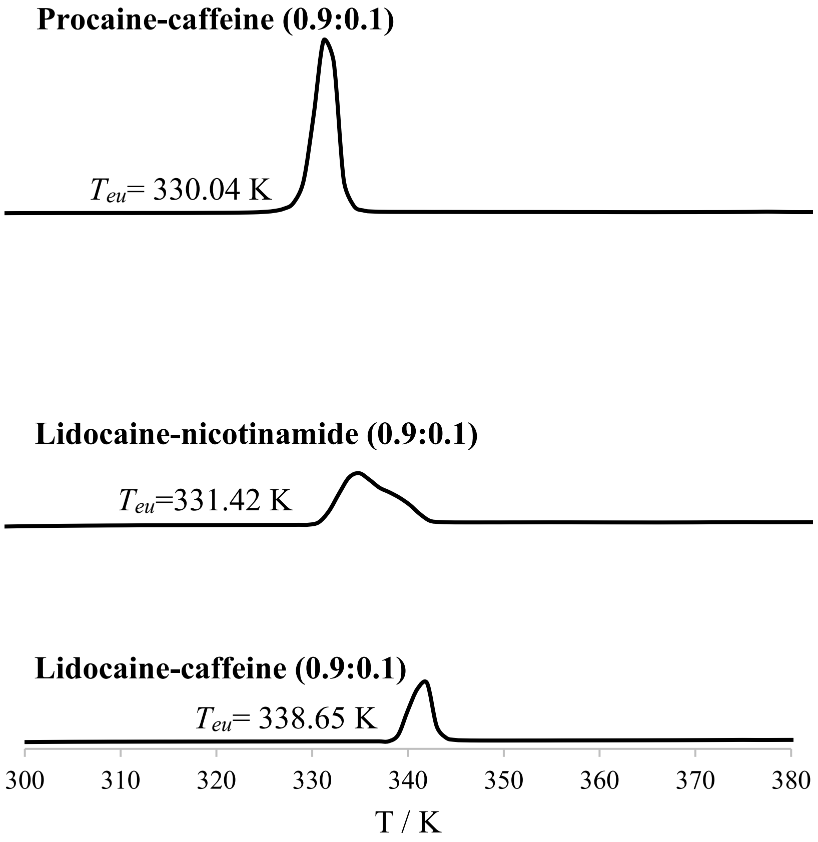

Figure S21. Differential scanning calorimetry curve of the API-hydrotrope binary mixtures at the eutectic compositions (mol API:mol hydrotrope).

Table S1. Melting temperature (T_m) and enthalpy (Δh_m) of the selected APIs and hydrotropes, measured in this work and compared to literature values.

**Compound**	**T_m/ K**		**Δh_m/ kJ mol^{-1}**	
	**This work**	**Literature**	**This work**	**Literature**
Benzocaine	362.32 ± 0.11	362.60[8]	25.03 ± 0.22	25.65[8]
Caffeine*	509.61 ± 0.00	510.20[8]	20.51 ± 0.00	20.08[8]
Lidocaine	340.86 ± 0.09	341.00[9]	18.75 ± 0.10	18.80[9]
Nicotinamide	401.72 ± 0.10	401.60[8]	25.32 ± 0.11	25.50[8]
Procaine	333.38 ± 0.11	335.00[9]	29.48 ± 0.11	26.22[9]
Urea	405.85 ± 0.00	406.20[8]	14.80 ± 0.00	14.60[8]
Water	-	273.00[10]	-	6.01[10]

* This work: T_{tr} = 417.20 K and the Δh_{tr} = 3.30 kJ mol^{-1}. From literature [8]: T_{tr} = 421.50 K and the Δh_{tr} = 3.29 kJ mol^{-1}

Table S2. Solid–liquid equilibria data of API-hydrotrope binary systems.

x_{API}	T_e/K	T^{liq}/K
Lidocaine + Urea		
0.1	338.65	409.75
0.2	338.65	409.15
0.3	338.75	408.35
0.4	338.75	407.95
0.5	338.75	407.85
0.6	338.85	407.95
0.7	339.35	407.15
0.8	339.55	407.25
0.9	407.85	407.85
Lidocaine + nicotinamide		
0.1	330.72	398.10
0.2	330.85	393.22
0.3	331.05	389.75
0.4	330.98	384.35
0.5	331.02	376.28
0.6	331.32	365.88
0.7	331.28	363.75
0.8	331.32	338.25
0.9	331.42	-
Lidocaine + caffeine		
0.1	338.65	503.85
0.2	338.65	489.95
0.3	338.58	481.22
0.4	338.65	469.95
0.5	338.65	460.12
0.6	338.65	443.90
0.7	339.02	421.95
0.8	338.75	339.02

0.9	338.65	-
0.1*	418.55*	-
0.2*	417.85*	-
0.3*	417.98*	-
0.4*	417.78*	-
0.5*	417.88*	-
0.6*	418.05*	-
Benzocaine + Urea		
0.1	358.35	408.25
0.2	358.95	408.45
0.3	359.25	407.65
0.4	359.55	407.95
0.5	359.45	405.95
0.6	359.55	406.25
0.7	359.55	406.25
0.8	359.45	406.35
0.9	359.25	405.85
Benzocaine + nicotinamide		
0.1	351.12	398.12
0.2	351.32	394.61
0.3	351.32	385.52
0.4	351.42	380.35
0.5	351.38	373.22
0.6	351.08	372.35
0.7	350.88	367.12
0.8	350.88	-
0.9	351.25	356.85
Benzocaine + caffeine		
0.1	351.95	502.55
0.2	351.92	486.02
0.3	351.92	473.18

0.4	351.78	465.25
0.5	351.78	448.35
0.6	351.75	416.15
0.7	351.72	393.58
0.8	350.88	-
0.9	351.05	358.55
0.1*	417.95*	-
0.2*	417.02*	-
0.3*	417.68*	-
0.4*	417.08*	-
0.5*	417.38*	-
0.6*	416.08*	-
Procaine + Urea		
0.1	332.05	409.85
0.2	332.25	409.65
0.3	332.35	408.85
0.4	332.35	408.15
0.5	332.75	407.45
0.6	332.55	407.65
0.7	332.55	407.75
0.8	332.55	407.05
0.9	332.55	407.05
Procaine + nicotinamide		
0.1	328.35	397.55
0.2	328.65	392.70
0.3	329.25	388.90
0.4	329.15	383.35
0.5	328.65	377.50
0.6	329.05	370.55
0.7	329.45	361.30
0.8	309.75	328.65

0.9	329.35	-
Procaine + caffeine		
0.1	330.25	498.55
0.2	330.25	491.45
0.3	330.25	488.15
0.4	330.15	480.25
0.5	330.25	471.15
0.6	330.35	440.55
0.7	330.45	414.35
0.8	330.55	393.75
0.9	330.65	-
0.1*	420.15*	-
0.2*	419.75*	-
0.3*	419.65*	-
0.4*	418.85*	-
0.5*	419.55*	-
0.6*	419.35*	-

* Solid-solid transition

The solubility data of APIs and hydrotropes in water

Tables S3 and S4 present the measured solubility data of the model APIs and selected hydrotropes in water, respectively, using the temperature-variant method.

Table S3. Measured solubility values of the APIs in water using the temperature variant method (n=3). The equivalent values in mg/mL are given in parentheses.

x_{API}	x_{water}	T/K
Lidocaine + water		
4.61244×10^{-5} (0.6)	0.999953876	304.15
9.99308×10^{-5} (1.3)	0.999900069	316.15
1.22989×10^{-4} (1.6)	0.999877011	322.15
Benzocaine + water		
1.30683×10^{-4} (1.2)	0.999869317	315.15
1.63986×10^{-4} (1.5)	0.999836014	318.15
2.39582×10^{-4} (2.2)	0.999760418	324.15
3.18332×10^{-4} (2.9)	0.999681668	330.15
Procaine + water		
6.89384×10^{-5} (0.9)	0.998695696	303.15
1.68398×10^{-4} (2.2)	0.997694467	315.15
2.14508×10^{-4} (2.8)	0.996795514	321.15

Table S4. Measured solubility values of the hydrotropes in water using the temperature variant method (n=3).

$x_{hydrotrope}$	x_{water}	T/K
Urea + water		
0.3000	0.7000	306.05
0.4000	0.6000	329.63
0.5000	0.5000	343.88
Nicotinamide + water		
0.0987	0.9013	297.10
0.1145	0.8855	301.05
0.1330	0.8670	305.65

0.1532	0.8468	310.85
0.1697	0.8303	314.60
0.1962	0.8038	324.18
0.2487	0.7513	335.97
0.2971	0.7029	343.28
Caffeine + water		
0.0166	0.9834	344.63
0.0124	0.9876	336.33
0.0086	0.9914	327.58
0.0027	0.9973	309.48
0.0051	0.9949	321.05

Urea-water binary SLE phase diagram

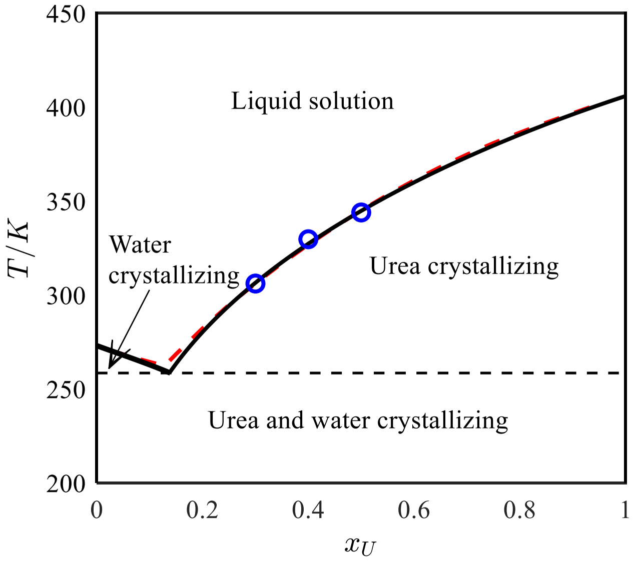

Figure S22. SLE phase diagrams of the urea-water system. Blue circles: liquidus temperatures; solid black line: NRTL-modeled liquidus (with the parameters from Table 3); dashed red line: NRTL-modeled liquidus (IUPAC[11]).

Binary SLE

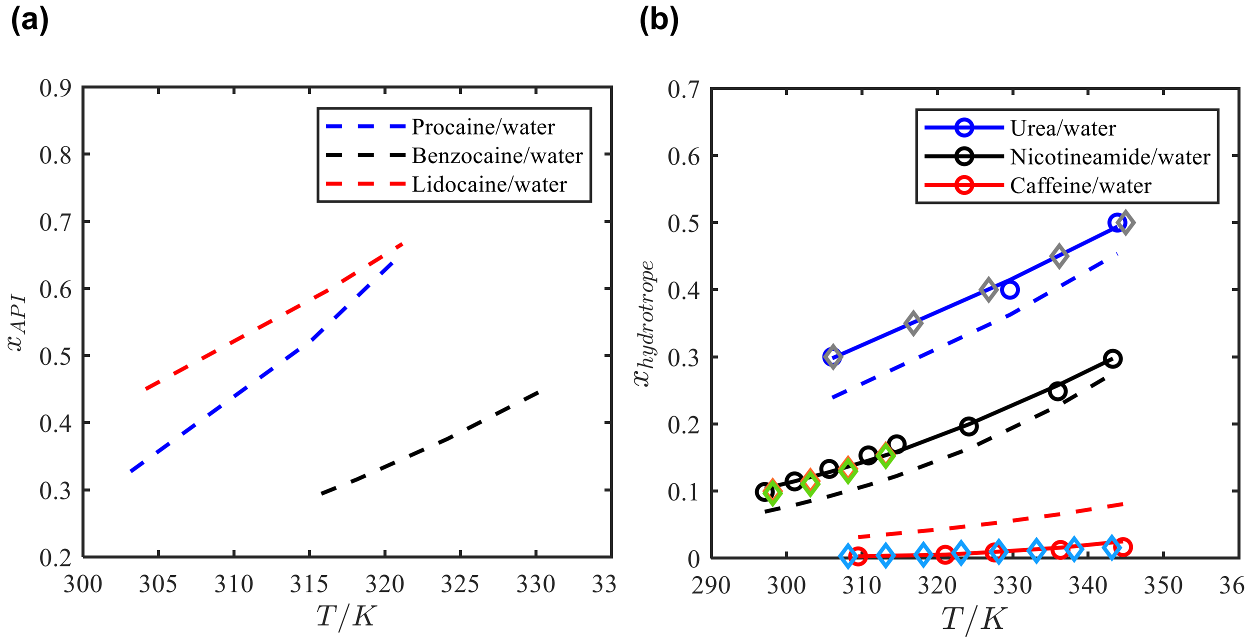

Figure S23. The solubility of (a) APIs in water at different temperatures calculated by the NRTL model and assuming ideal solution behavior and (b) hydrotropes in water at different temperatures. Circles – experimental data (this work); Diamonds – experimental data from literature (gray [11], orange [12], green [13] and light blue [14]); lines – solubility calculated with the NRTL model; dashed lines – ideal solubility lines.

Ternary SLE phase diagrams at 310 K

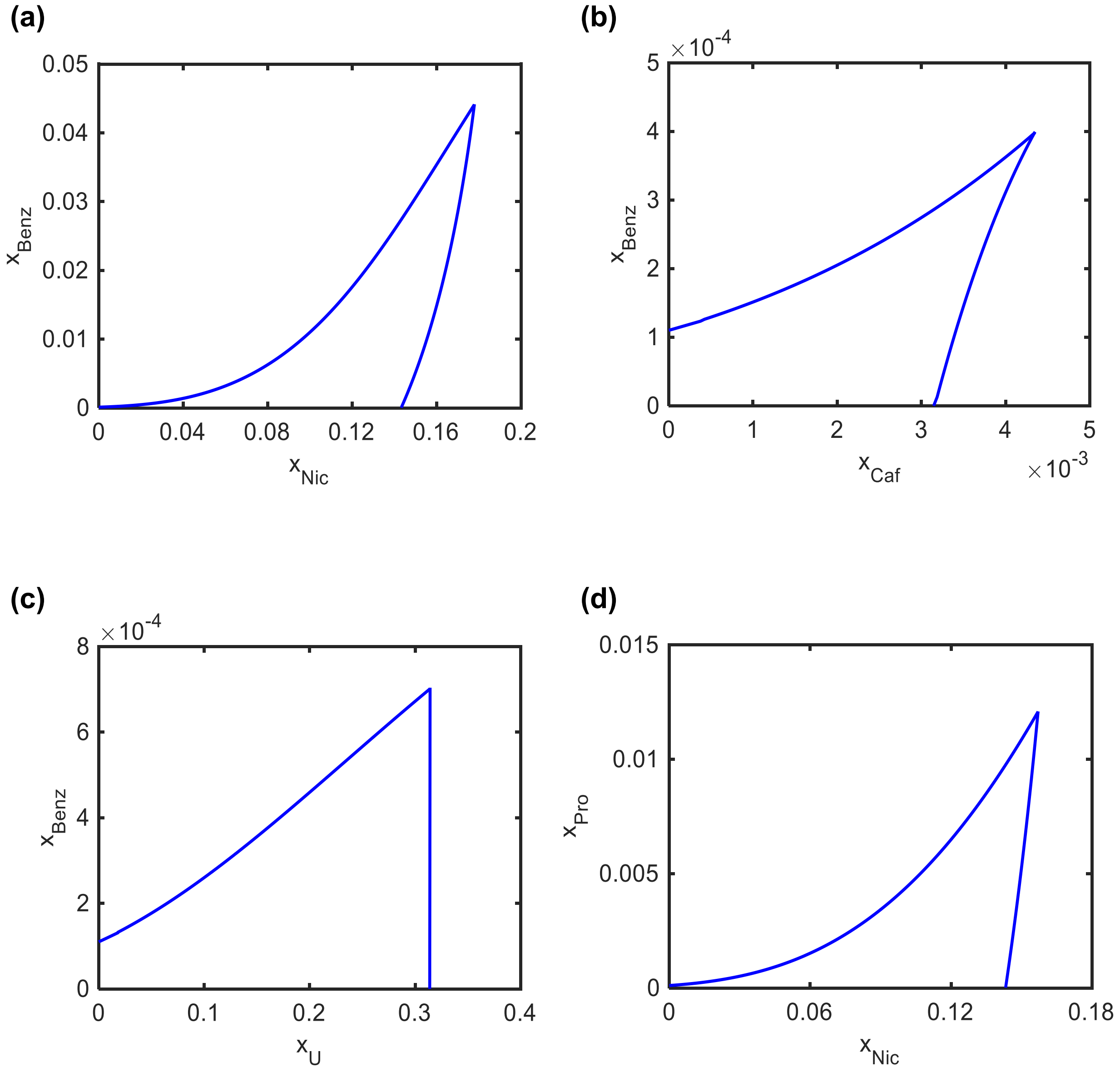

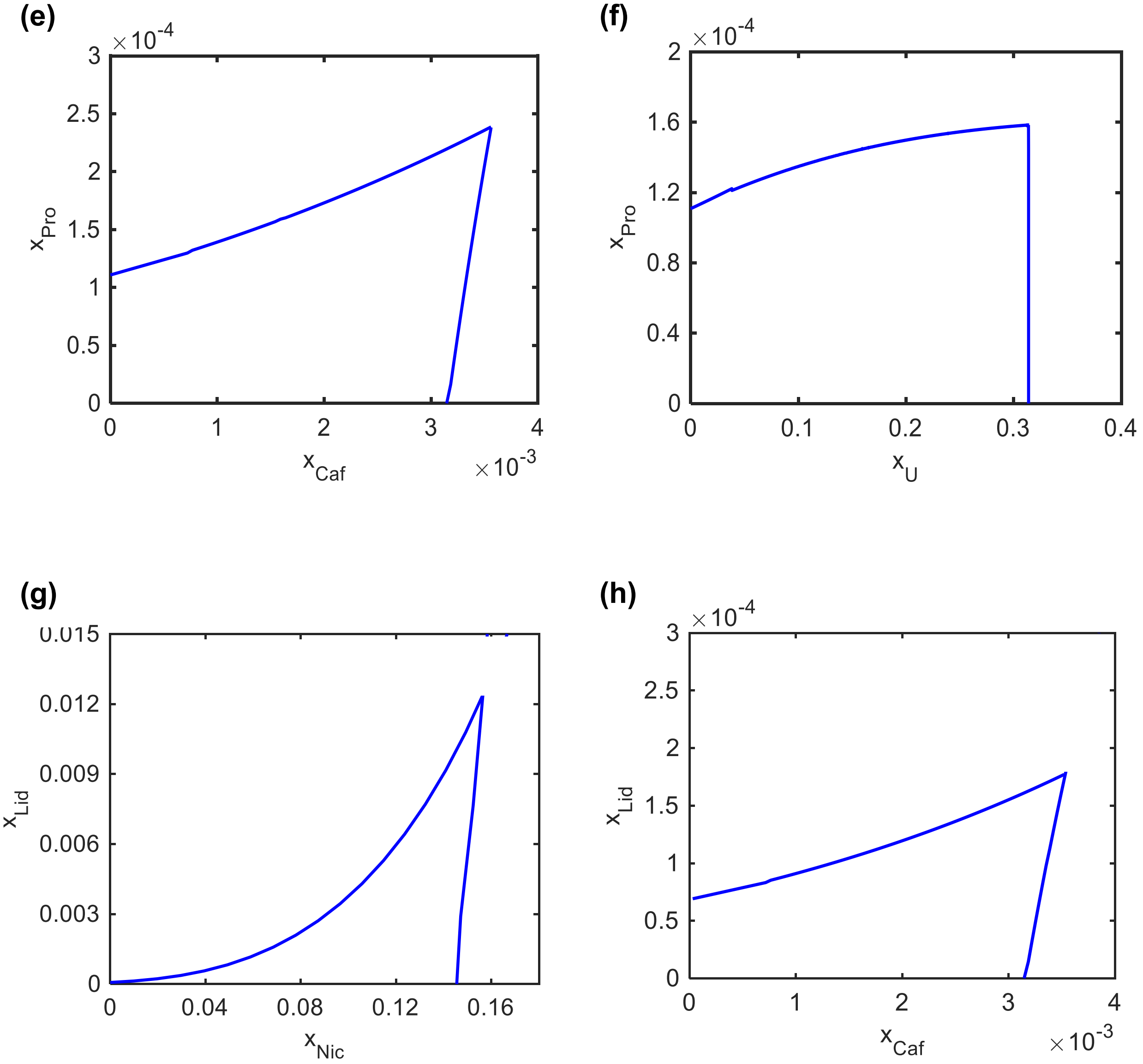
(e)
×10^-4
3
2.5
2
1.5
1
0.5
0
x_Pro
0
1
2
3
4
x_Caf
×10^-3
(f)
×10^-4
2
1.6
1.2
0.8
0.4
0
x_Pro
0
0.1
0.2
0.3
0.4
x_U
(g)
0.015
0.012
0.009
0.006
0.003
0
x_Lid
0
0.04
0.08
0.12
0.16
x_Nic
(h)
×10^-4
3
2.5
2
1.5
1
0.5
0
x_Lid
0
1
2
3
4
x_Caf
×10^-3

(i)

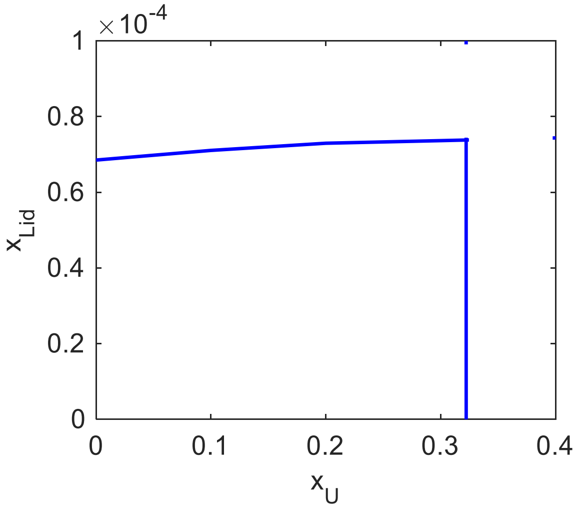

Figure S24. Calculated SLE phase diagrams of the nine API-hydrotrope-water studied systems at 310 K with the NRTL model. (a) benzocaine-nicotinamide-water, (b) benzocaine-caffeine-water, (c) benzocaine-urea-water, (d) procaine-nicotinamide-water, (e) procaine-caffeine-water, (f) procaine-urea-water, (g) lidocaine-nicotinamide-water, (h) lidocaine-caffeine-water, and (i) lidocaine-urea-water.

REFERENCES

(1) Sinha, B. K.; Pattabhi, V. Crystal structure of benzocaine—A local anaesthetic. *Journal of Chemical Sciences* **1987**, *98*, 229-234.

(2) Lehmann, C. W.; Stowasser, F. The crystal structure of anhydrous β-caffeine as determined from X-ray powder-diffraction data. *Chemistry–A European Journal* **2007**, *13* (10), 2908-2911.

(3) Bambagiotti-Alberti, M.; Bruni, B.; Di Vaira, M.; Giannellini, V.; Guerri, A. 2-(Diethylamino)-N-(2, 6-dimethylphenyl) acetamide, a low-temperature redetermination. *Structure Reports* **2007**, *63* (2), o768-o770.

(4) Wright, W.; King, G. The crystal structure of nicotinamide. *Acta Crystallographica* **1954**, *7* (3), 283-288.

(5) Kashino, S.; Ikeda, M.; Haisa, M. The structure of procaine. *Structural Science* **1982**, *38* (6), 1868-1870.

(6) Swaminathan, S.; Craven, B. M.; McMullan, R. K. The crystal structure and molecular thermal motion of urea at 12, 60 and 123 K from neutron diffraction. *Structural Science* **1984**, *40* (3), 300-306.

(7) Chan, E. J.; Rae, A. D.; Welberry, T. R. On the polymorphism of benzocaine; a low-temperature structural phase transition for form (II). *Structural Science* **2009**, *65* (4), 509-515.

(8) Acree, W.; Chickos, J. S. Phase transition enthalpy measurements of organic and organometallic compounds. Sublimation, vaporization and fusion enthalpies from 1880 to 2015. Part 1. C1− C10. *Journal of Physical and Chemical Reference Data* **2016**, *45* (3).

(9) Acree, W.; Chickos, J. S. Phase transition enthalpy measurements of organic and organometallic compounds and ionic liquids. Sublimation, vaporization, and fusion enthalpies from 1880 to 2015. Part 2. C11–C192. *Journal of Physical and Chemical Reference Data* **2017**, *46* (1).

(10) Lide, D. R. *CRC handbook of chemistry and physics*; CRC press, 2004.

(11) Bazyleva, A.; Acree, W. E.; Diky, V.; Hefter, G. T.; Jacquemin, J.; Magalhães, M. C. F.; Magee, J. W.; Nordstrom, D. K.; O'Connell, J. P.; Olson, J. D.; et al. Reference materials for phase equilibrium studies. 2. Solid–liquid equilibria (IUPAC Technical Report). **2022**, *94* (11-12), 1225-1247.
(12) Cysewski, P.; Przybyłek, M.; Kowalska, A.; Tymorek, N. Thermodynamics and intermolecular interactions of nicotinamide in neat and binary solutions: Experimental measurements and COSMO-RS concentration dependent reactions investigations. *International Journal of Molecular Sciences* **2021**, *22* (14), 7365.
(13) Wu, H.; Dang, L.; Wei, H. Solid–liquid phase equilibrium of nicotinamide in different pure solvents: Measurements and thermodynamic modeling. *Industrial & Engineering Chemistry Research* **2014**, *53* (4), 1707-1711.
(14) Dabir, T. O.; Gaikar, V. G.; Jayaraman, S.; Mukherjee, S. Thermodynamic modeling studies of aqueous solubility of caffeine, gallic acid and their cocrystal in the temperature range of 303 K–363 K. *Fluid Phase Equilibria* **2018**, *456*, 65-76.
